# Kynurenine Pathway Dysregulation Impairs Podocyte Morphology and Bioenergetics In Vitro and Leads to Glomerular Dysfunction

**DOI:** 10.1096/fj.202502175R

**Published:** 2025-11-12

**Authors:** Patricia Bolanos‐Palmieri, Heiko Schenk, Heike Bähre, Patricia Schroder, Lynne Staggs, Hermann Haller, Mario Schiffer

**Affiliations:** ^1^ Department of Nephrology and Hypertension Friedrich‐Alexander‐Universität Erlangen‐Nürnberg Erlangen Germany; ^2^ Department of Nephrology and Hypertension Hannover Medical School Hannover Germany; ^3^ Research Core Unit Metabolomics Hannover Medical School Hannover Germany; ^4^ Mount Desert Island Biological Laboratory Salisbury Cove Maine USA

**Keywords:** cytoskeleton, kynurenine, kynurenine 3‐monooxigenase, mitochondria, podocyte, proteinuria

## Abstract

Managing tryptophan (TRP) availability is important for cell homeostasis, and a dynamic balance between dietary intake and its catabolism is crucial. The enzymes of the kynurenine pathway (KP) mediate the main catabolic route for TRP. Its intermediary products, collectively known as kynurenines, are considered metabolically active and highly pleiotropic. Some progress has been made in the description of the biological function of the kynurenines, and despite the growing number of studies that show an association between TRP metabolism and kidney function, not much is known about the cellular mechanisms involved. To assess if the kynurenines play a role in glomerular dysfunction, we carried out a series of experiments aimed at describing the effect of changes in the relative abundance of the kynurenines on cells of the glomerulus, both in vivo and in vitro. We used a transgenic zebrafish line as a model to show that systemic changes in the KP either by morpholino knockdown, enzymatic inhibition, or kynurenine supplementation, lead to pericardial effusion, yolk sac edema, and excretion of high molecular weight proteins, all signs of impaired glomerular filtration. Cultured podocytes incubated with a KP inhibitor show changes in cell size, morphology and focal adhesions, leading to a higher detachment rate. Additionally, there is a change in the polarization status of the mitochondria, showing a loss of membrane potential and an alteration of bioenergetics parameters. Taken together, our results highlight the importance of kynurenine metabolite levels in the maintenance of a functioning filtration barrier.

## Graphical abstract

The enzymes of the kynurenine pathway participate in the catabolism of TRP, where they generate metabolites known as kynurenines, which are considered biologically active. Using a transgenic zebrafish line, we showed that systemic changes in the kynurenine pathway lead to edema and proteinuria, both signs of an impaired glomerular filtration barrier. Additionally, inhibition of the pathway in cultured podocytes resulted in alterations to the cytoskeleton, increased cell detachment, and changes in redox status and mitochondrial function.

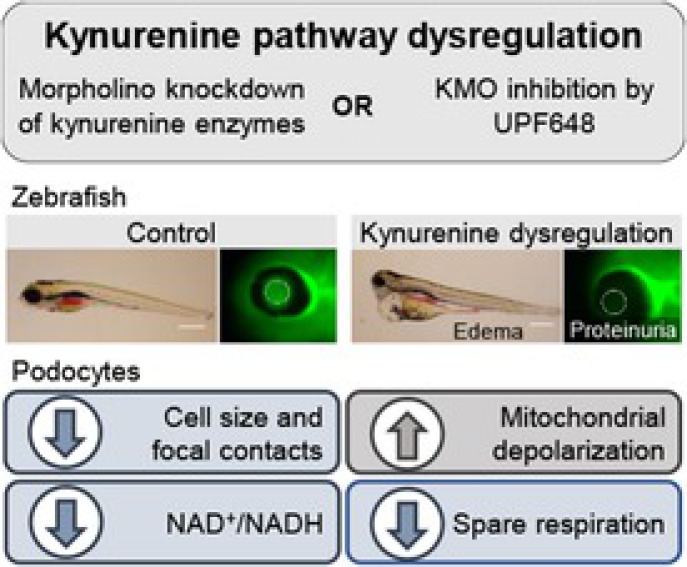

## 1 Introduction

**FIGURE 1:**
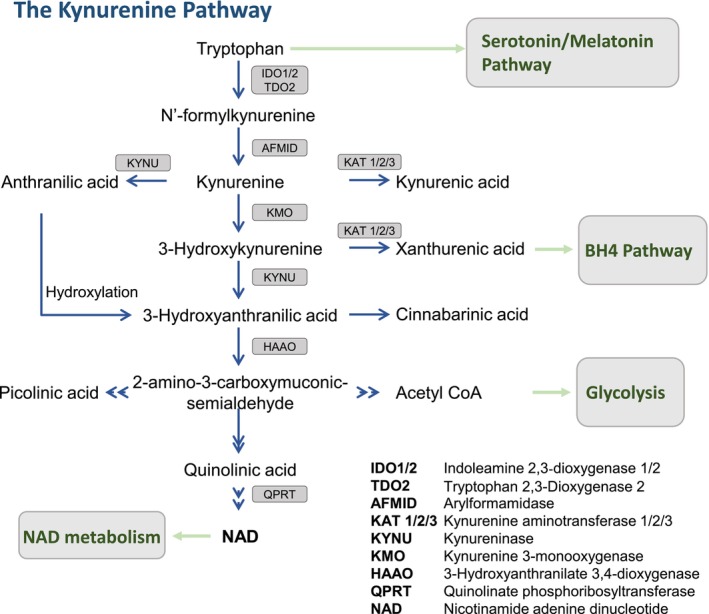
Simplified schematic representation of the kynurenine pathway. Catabolism through the kynurenine pathway is the major route for tryptophan degradation which results in the production of a series of bioactive metabolites. The kynurenine metabolites themselves participate in many signaling pathways and their dysregulation has been shown to contribute to both cell homeostasis as well as disease etiology.

Tryptophan (TRP) is an essential amino acid that, apart from being crucial for protein synthesis, is the precursor of a series of bioactive compounds that are largely pleiotropic and influence a wide array of cell processes [1], including the *de novo* synthesis of the coenzyme nicotinamide adenine dinucleotide (NAD) [2, 3]. Four pathways are involved in the catabolism of TRP, namely transamination (generates indole‐3 pyruvic acid), hydroxylation (production of serotonin and melatonin), decarboxylation (produces tryptamine) and oxygenation (production of kynurenine metabolites and NAD). Of these, the kynurenine pathway (KP) represents the main route for TRP degradation (Figure 1) [4, 5, 6], and alongside dietary intake, it contributes to the regulation of systemic TRP stores. Since both the excess and depletion of TRP and its metabolites have been shown to be detrimental to health, proper regulation of the KP is vital (reviewed in [7, 8]). Although the majority of TRP catabolism happens in the liver and the intestines [7, 9], a growing body of evidence has highlighted the impact of the KP on the kidneys, as well as the importance of the kidneys in the kynurenine (KYN) metabolite clearance from the circulation. The kidneys possess an extensive enzymatic machinery capable of responding to the biologically active KYN metabolites; because of this, an impaired modulation of the KP, both systemically and locally in the kidneys, could have important clinical and therapeutic implications (reviewed in [10, 11]).

Data from animal models suggest that changes in enzyme expression levels or knockout of specific KYN enzymes impact acute kidney injury [12], multiorgan failure (including the kidney) [13], fatal kidney failure [14], and the development of proteinuria [15, 16]. Moreover, several studies have linked the increase in activity of the KP, marked by elevated levels of KYN and its metabolites in the circulation, with the onset and severity of chronic kidney disease (CKD), as well as with associated comorbidities like changes in bone density [17], muscle loss [18], endothelial dysfunction and cardiovascular events [19, 20, 21], an increase in inflammation [22] and alterations in behavior (e.g., CKD‐associated fatigue) [23]. Additionally, circulating levels of kynurenine and kynurenic acid (KYNA) have been consistently correlated with disease progression in patients with renal insufficiency undergoing hemodialysis [21, 24], with the progression to end‐stage renal disease in cases of type 2 diabetes associated CKD [25], allograft rejection after kidney transplantation [26, 27], and in animal models of experimental renal failure [28, 29]. Consistent with these results, baseline serum KYN/TRP ratio levels have also been associated with incident CKD and a change in kidney function indicated by a decline in eGFR [30]. One of the key regulators of KYN availability, other than the catabolic pathway activation, is the mitochondrial enzyme Kynurenine‐3‐monooxygenase (KMO). The kidney and the liver possess the highest KMO catalytic activity [31], making them attractive targets for additional research.

Although there is an ever‐increasing body of evidence that shows an association between systemic levels of TRP catabolic products and kidney function, and great advances have been made in the investigation of the KP as a druggable target in diverse pathologies, there is still a lot left to explore in terms of the cellular mechanisms that respond to the varying metabolite concentrations. Additionally, it is still not entirely clear whether the changes in serum metabolites are a cause or a consequence of a decline in kidney function. Cheng et al. 2020 showed that KYN, alongside three other TRP metabolites, appears to increase in the circulation as a consequence of reduced eGFR, implying there might not be a direct causal relationship with the decline in kidney function [32]. However, the high pleiotropy of the pathway and its intermediate metabolites suggest that the physiological significance of the KYNs will continue to be a subject of research. This is especially true when considering how changes in KP enzymes and metabolites impact renal cells and disease progression.

In the kidney, our laboratory is particularly interested in the cells that reside inside a complex structure that functions as a dynamic macromolecular sieve called the glomerulus. The glomerulus comprises the podocytes, mesangial and endothelial cells, and is surrounded by a monolayer of parietal epithelial cells that encase the glomerular tuft within the urinary space [33]. The adequate filtration of the blood in the glomerulus is reliant upon a healthy filtration barrier, which is made up of three distinct layers: the fenestrated endothelium, the glomerular basement membrane, and the slit diaphragm formed by the foot processes of the podocytes [34, 35, 36]. The podocytes are highly specialized cells that have a distinct architecture linked to a dynamic and tightly regulated cytoskeleton, which provides a structural and signaling scaffold to respond to a microenvironment in constant flux [37, 38, 39, 40].

In this work, we aim to describe the repercussions of KP dysregulation, specifically on glomerular cells, both in vivo using a zebrafish model and in vitro through cultured human and murine glomerular epithelial cells. To achieve this, we explored the consequences of KP downregulation using morpholinos in zebrafish larvae, focusing particularly on indicators of kidney function. Additionally, we examined the effects of KMO inhibition on cell morphology, substrate adhesion, mitochondrial function and cell bioenergetics in vitro.

## 2 Materials and Methods

### 2.1 Materials and Antibodies

Inhibition of the KP was carried out using UPF648 (Tocris Bioscience, Bristol, UK), a KMO inhibitor that binds to the FAD domain and modifies the active site, which essentially blocks the substrate (kynurenine) from binding to the enzyme [41]. A stock solution at a concentration of 1 mM was prepared in ethanol, as specified by the manufacturer. In the experiments where KYN was supplemented in the media, we used a stock solution of 10 mM L‐kynurenine (Sigma, St. Louis, Missouri, USA) prepared in DMSO (Roth, Karlsruhe, Germany). Working dilutions were then prepared either in cell culture media or zebrafish media, depending on the experiment. The concentrations used in each experiment, as well as the length of the incubation, are detailed in each figure in the results section.

To visualize focal contacts and actin filaments, we used an antibody against Paxillin (Merk‐Millipore, Burlington, MA, USA) and fluorescently labeled Phalloidin (Thermo Fisher Scientific, Waltham, MA, USA). All nuclei were marked with DAPI (Thermo Fisher Scientific, Waltham, MA, USA), and those nuclei that belonged to actively proliferating cells were stained with an antibody against Ki‐67 (Abcam, Cambridge, UK). Kynurenine 3‐monooxygenase expression in cells was assessed using an anti‐KMO antibody (Proteintech, Rosemont, IL, USA). Mitochondria were visualized using MitoTracker Orange CMTMRos (Invitrogen, Waltham, MA, USA) following cell fixation, as per the manufacturer's instructions. All reagents used were of analytical grade and stored and handled according to the safety guidelines provided by the manufacturers.

### 2.2 In Vivo Zebrafish Studies

The in vivo experiments were carried out using transgenic zebrafish ( *Danio rerio* Tg[l‐fabp:eGFP‐DBP]) embryos [42]. Animals were housed and mated at 28.5°C and embryos were handled in standard Embryo Raising Media (ERM): (1X solution: MgSO_4_·7H_2_O [1 mM], Na_2_HPO_4_·7H_2_O [0.05 mM], KH_2_PO_4_ [0.15 mM], CaCl_2_·2H_2_O [1 mM], KCl [0.5 mM], NaCl [15 mM]). The MDI Biological Laboratory (Bar Harbor, ME, USA) animal care committee approved the animal protocol (IACUC protocol #1703).

#### 2.2.1 Morpholino‐Mediated Knockdown of Enzymes of the KP in Zebrafish

For morpholino (MO) knockdown experiments, fertilized eggs were randomly assigned to either MO injection or control groups and 4.6 nL of each MO diluted in injection buffer (HEPES [20 mM], KCl [200 mM], and phenol red 8 [0.75%]) were injected into the yolk of fertilized eggs at the 1–4 cell stage using the Nanoject II injection device (Drummond Scientific, Broomall, PA, USA) Morpholinos (MO) were injected in the following concentrations: Ido1 200 μM, Tdo2a 200 μM, Tdo2b 200 μM, Afmid 30 μM, Kmo 100 μM, Kynu 30 μM, Haao 150 μM. Optimal MO concentrations were selected by determining which one achieves a balance between phenotype development and larval mortality. Morpholino sequences targeting the enzymes of the KP were designed and ordered from GeneTools (Philomath, OR, USA) (Table 1). Knockdown of the KYN enzymes was confirmed with the metabolomics analysis (by accumulation of upstream metabolites) or with qPCR, where possible (Figure S3).

**TABLE 1:** List of morpholinos used to knock down the expression of selected enzymes of the kynurenine pathway.

Target gene	Morpholino sequence	Type
Ido1	5′‐CGCATAAGTCATTTACCCATTTGGA‐3′	Splice donor/acceptor
Tdo2a	5′‐TAGTATTTGCTCTACTCACATGTCC‐3′	Splice donor/acceptor
Tdo2b	5′‐ATTGAAGGTTGAGTTATTTACCTGT‐3′	Splice donor/acceptor
Afmid	5′‐CACAGAGGAGGAGAAACAGACCTAA‐3′	Splice donor/acceptor
Kmo	5′‐GATGTGAGAAAGCTGTCTCCATGTT‐3′	ATG blocker
Kynu	5′‐ATCCGCTCCATCATCTCCACAGAAC‐3′	ATG blocker
Haao	5′‐TTAAATGATTCTTACCTCACCTTCT‐3′	Splice donor/acceptor
Scrambled control	5′‐CCTCTTACCTCAGTTACAATTTATA‐3′	—

#### 2.2.2 qPCR Quantification of KYN Enzymes

mRNA was extracted from zebrafish larvae harvested at 120 hpf with the RNAeasy Minikit (Qiagen, Hilden, Germany) following the manufacturer's instructions. 1 μg RNA per sample was retrotranscribed, using Oligo(dT)primers (Promega, Madison, WI, USA), random primers (Promega, Madison, WI, USA), dNTPs (Roche, Mannheim, Germany), and M‐MLV reverse transcriptase and appropriate buffer (Promega, Madison, WI, USA). The qPCR was carried out using fast start Taq polymerase with SYBR green as the intercalating dye (Invitrogen). Real‐time PCR was carried out using the Light Cycler 480 thermocycler system (Roche). The values were normalized to Hprt and presented as fold change relative to controls. The zebrafish primers are as follows:

Ido1 forward—TTGGGTCTGCCACCAATCC, reverse—CAATGGTGAAGGGACCATTTG.

Afmid forward—ACGTGCCGCTGGTGATTTAT, reverse—TCCATATCACCTTTCGGAGCG.

Hprt forward—ACCAAAACACTATGCGGCTG, reverse—GTGTCCACCCATGTGCTTCA.

#### 2.2.3 Phenotype Evaluation of Morphant Fish

Following the morpholino injection, the fish embryos were monitored over 96 h. To ensure that morpholinos do not have large negative effects on cardiac development that could impact the formation of a glomerular capillary tuft, the presence or absence of blood flow was monitored in the tail region of the larvae at 48 hpf; those fish with no or very slow blood flow at this stage were eliminated from the experiment. The presence of blood flow in the larvae is monitored until the end of the experiment [43]. At 96 hpf, the larvae were categorized depending on the severity of the yolk sac edema and pericardial effusion by visual inspection, where P1 = no edema, P2 = moderate edema, P3 = severe edema and P4 = very severe edema, as was previously described [43]. To assess glomerular filtration barrier integrity and determine if MO‐knockdown leads to proteinuria, transgenic Tg(l‐fabp:eGFP‐DBP) zebrafish were used. This fish line contains a transgenic construct (eGFP‐DBP) that codes for a green fluorescent protein tagging the vitamin D binding protein. This construct is comparable in size to human albumin and accumulates in the circulatory system of the fish larva if the glomerular filtration barrier remains intact. On the other hand, when the filtration barrier is compromised, the fluorescence signal within the circulation is reduced as proteins are excreted through the injured glomerulus. To determine if the glomerular filter is working, we used a well‐established assay that uses live microscopy to monitor circulating eGFP‐DBP; fluorescence intensity levels are then measured with ImageJ (Version 1.51a, National Institutes of Health, Bethesda, MD, USA [44]) based on gray‐scale images of the retinal vessel plexus of the larvae, acquired using a fluorescence microscope and reported in arbitrary units (AU). The retinal vessel plexus is used because it is a convenient way to reproducibly estimate changes in fluorescence intensity within a defined area in the fish's circulatory system, which correlates with a loss of integrity of the glomerular filtration barrier after injury, as we have previously described [45].

#### 2.2.4 Kmo Inhibition and KYN Supplementation in Zebrafish

The embryos were monitored for clutch quality and development for 48 h postfertilization (hpf); at this point, the larvae were distributed in separate groups, each including 10–15 fish, and were treated with the relevant compounds UPF648 (Tocris Bioscience, Bristol, UK; 50 and 100 μM), Kynurenine (Sigma, St. Louis, Missouri, USA; 10, 50 and 100 μM), a combination of both, or ethanol and DMSO controls, for 48 h via immersion treatment in the ERM. Phenotype and proteinuria readouts were taken after the treatment, as described for morpholino knockdown experiments in the previous section.

### 2.3 Glomerular Cell Culture

Cell culture conditions for human [46] and mouse [47] immortalized podocytes have been described elsewhere. Briefly, proliferating, undifferentiated cells were kept at 33°C in a humidified 5% CO_2_ incubator in RPMI‐1640 media with stable L‐Glutamine (Biochrom AG, Cambridge, UK), supplemented with 10% FBS (Gibco, Waltham, MA, USA) and 1% Penicillin/Streptomycin (Invitrogen, Waltham, MA, USA). During the proliferation stage, mouse podocyte media was additionally supplemented with 10 U/mL of interferon‐gamma (INF‐γ, Cell Sciences, Newburyport, MA, USA) and the cells were kept in cell culture flasks coated with Collagen Type I (BD Bioscience, Franklin Lakes, NJ, USA). The human podocytes receive insulin supplementation (Sigma, St. Louis, Missouri, USA) at a concentration of 10 μg/mL. The cells were passaged when they reached 80%–90% confluence and seeded according to the experimental requirement. Undifferentiated human podocytes were seeded at a density of 0.35–0.42 × 10^4^ cells/cm^2^; undifferentiated mouse podocytes at 0.37–0.45 × 10^4^ cells/cm^2^. Before starting the experiments, the cells were allowed to differentiate in a 37°C humidified 5% CO_2_ incubator in complete RPMI media for 7–14 days. During this period, the media was changed every other day. Mouse parietal epithelial cells were cultured in RPMI‐1640 media with 10% FBS and 1% Penicillin/Streptomycin in a humidified incubator at 37°C and 5% CO_2_, as was previously described [48] and seeded at a density of 0.20–0.25 × 10^4^ cells/cm^2^.

#### 2.3.1 In Vitro Experiments With Glomerular Cells

Human podocytes, mouse podocytes, or mouse parietal epithelial cells were seeded onto glass cover slides or Nunclon Delta tissue culture‐treated plates (Thermo Fisher Scientific, Waltham, MA, USA) and allowed to differentiate for 7–10 days. They were then treated with either the KMO inhibitor UPF648 (Tocris Bioscience, Bristol, UK) or with the vehicle control. Concentrations and time points varied according to the type of experiment, and they are specified in the figure legends of each result section.

#### 2.3.2 Immunofluorescence Staining

To visualize intracellular structures, the adherent cells were washed with PBS and fixed in 4% PFA (Roth, Karlsruhe, Germany) for 15 min at room temperature. The cells were then washed 2 times with PBS and permeabilized using 0.1% Triton X‐100 (Sigma, St. Louis, Missouri, USA). After another PBS wash, nonspecific antibody binding was blocked with 10% donkey serum (Sigma, St. Louis, Missouri, USA) for 30 min. Primary antibody incubation (1:50) was carried out overnight at 4°C and the appropriate fluorophore‐coupled secondary antibody (1:500) was used the next day for a 1 h incubation at room temperature. Cells were washed with 1X PBS, mounted using Fluoromount with DAPI (Thermo Fisher Scientific, Waltham, MA, USA), and stored at 4°C awaiting sample imaging and analysis.

#### 2.3.3 Quantification of Cell Area

At the end of the KMO inhibition period, the cells were fixed with 4% PFA (Roth, Karlsruhe, Germany) and then stained with phalloidin to help visualize the cell shape and structure. Staining was visualized and photographed using an inverted Leica DMLB microscope coupled to a Leica DFC425C camera. 8‐Bit images were acquired as TIFF files. To determine if there are changes in cell area as a result of KMO inhibition, the outline of each of the cells in a field of view was traced, and their area was measured using ImageJ (Version 1.51a, National Institutes of Health, Bethesda, MD, [44]) and reported in μm^2^.

#### 2.3.4 Caspase 3/7 Activation Apoptosis Assay

Mouse and human podocytes were seeded in 96‐well cell culture plates and differentiated as described above. Cells were then treated with UPF648 or ethanol control, and caspase activation was measured using the Caspase‐Glo 3/7 Assay (Promega, Madison, WI, USA), following the manufacturer's protocol. Luminescence was detected using a Synergy HT multidetection microplate reader (BioTek, Winooski, VT, USA).

#### 2.3.5 Mitochondrial Membrane Potential

To assess the impact of KMO inhibition on the mitochondrial membrane potential, mouse glomerular cells were seeded in 96‐well plates with dark sides and clear bottoms (Thermo Fisher Scientific, Waltham, MA, USA). The cells were then treated with UPF648 or control in phenol red‐free RPMI media, supplemented as described previously for passaging cells. JC‐1 Mitochondrial Membrane Potential Assay Kit (Abcam, Cambridge, UK) was used to determine the polarization status following the manufacturer's protocol. This contains a cationic dye that emits a signal at 590 nm when present in high concentrations inside mitochondria with high membrane potential; otherwise, in depolarized mitochondria, it emits at 530 nm. Fluorescence was analyzed in a Synergy HT multi‐detection microplate reader (BioTek, Winooski, VT, USA) and the cells' polarization status is presented as a ratio between aggregate and monomeric dye (Em 590/Em 530). The mitochondrial decoupler, FCCP, was used as a positive control for mitochondrial depolarization. To show that the dye works as expected, a pilot experiment was carried out where mouse podocytes were treated with UPF648 and images at 530 nm and 590 nm were acquired using an inverted Leica DMLB microscope coupled to a Leica DFC425C camera; representative images of the individual channels, as well as a merged image, are shown in Figure S7.

#### 2.3.6 NAD+/NADH Ratio

After KMO inhibition in the podocytes by treatment with UPF648 the redox status of NAD was assessed using the NAD/NADH‐Glo Assay (Promega, Madison, WI, USA) following the modified protocol provided by the manufacturer for independent determination of NAD+ or NADH from one sample, based on the differential stability in acid or basic pH. The luminescence signal was detected using a Synergy HT multi‐detection microplate reader (BioTek, Winooski, VT, USA). Data are presented as a ratio of the luminescence from the acid‐treated samples to the base‐treated samples (NAD+/NADH).

#### 2.3.7 Microplate‐Based Bioenergetic Profiling

To determine if KMO inhibition leads to changes in mitochondrial function, we used the Mito Stress test and the Seahorse XF Extracellular Flux Analyzer (both from Agilent, Santa Clara, CA, USA) following the protocol provided by the manufacturer. In short, after podocyte differentiation and before the addition of UPF648, the cells were trypsinized and 5.0 × 10^4^ cells per well were seeded onto collagen‐coated XF24 VC‐treated polystyrene plates (Agilent, Santa Clara, CA, USA) and allowed to attach overnight. As instructed in the protocol, 4 wells in each plate were left without cells and used as background correction controls. For the measurements, assay media was prepared fresh per the manufacturer's instructions: nonbuffered RPMI media (Agilent, Santa Clara, CA, USA) with 1 mM pyruvate, 2 mM glutamine, and 10 mM glucose. pH was adjusted to 7.4 and then filter‐sterilized. Prehydrated XF24 sensor cartridges were loaded with the compounds to deliver injections as follows: Port A: Oligomycin (ATP synthase inhibitor) final concentration 2.5 μM. Port B: FCCP (mitochondrial uncoupler) final concentration 2 μM, and Port C: Rotenone and Antimycin A (block mitochondrial respiration by targeting complex I and III) at a final concentration of 0.5 μM. The sequential addition of these compounds while simultaneously measuring the oxygen consumption rate (OCR) allows for the calculation of a range of bioenergetic parameters using the Seahorse XF Mito Stress Test Report Generator (Figure S1).

### 2.4 Mass Spectrometry of KYN Metabolites

Zebrafish larvae were collected at 120 hpf or 96 hpf after morpholino injection or UPF648 treatment, respectively. 10–15 larvae from each group were anesthetized with tricaine (Sigma, St. Louis, Missouri, USA) and placed on ice for 20 min until cessation of heartbeat. Immediately afterwards, they were placed in Safe‐Lock 1.5 mL tubes (Eppendorf, Hamburg, Germany), all the ERM was removed from the fish, and they were flash‐frozen in liquid nitrogen. Each sample was then resuspended in 800 μL of ice‐cold extraction solvent composed of acetonitrile/methanol/water (2/2/1, v/v/v; J.T. Baker, Waltham, MA, USA) and transferred to a FastPrep tube containing Lysing Matrix D (1.4 ceramic spheres, MP Biomedicals, Santa Ana, CA, USA) and homogenized using a FastPrep24 cell and tissue homogenizer (5 m/s, 30 s; MP Biomedicals, Santa Ana, CA, USA). The lysates were then heated at 95°C for 10 min and the proteins were precipitated by freezing at −80°C overnight. Protein removal was done by centrifugation (10 min at 4°C, 14000 rpm). For subsequent metabolite quantification, 320 μL of the sample supernatant was evaporated to dryness at 40°C in a Speed‐Vac vacuum concentrator. The samples were then resuspended in 150 μL of water (HPLC grade) with 0.1% formic acid and 1.33 μM caffeic acid, as internal standards. Metabolite quantification was done via HPLC‐MS/MS using a QTrap5500 triple quadrupole mass spectrometer (SCIEX). LLOQ (lower level of quantification) was 0.369 pmol/sample for TRP, 0.133 pmol/sample for kynurenic acid, 0.147 pmol/sample for KYN, 2.81 pmol/sample for NAD+ and 0.0676 pmol/sample for 3‐hydroxykinurenine.

In the case of the glomerular cells, after the KMO inhibition by UPF648 the cells were washed with cold PBS, and analyte extraction and protein denaturation were carried out in 300 μL of cold extraction solvent (acetonitrile/methanol/water in a ratio of 2/2/1, v/v/v; J.T. Baker, Waltham, MA, USA) with 0.665 μM caffeic acid as an internal standard (final sample concentration = 1.33 μM). Two subsequent washes of the cell culture well and the cell scraper were done with the extraction solvent without the internal standard and their volume was added to that of the cell lysate. Samples were vortexed for 30 s and protein precipitation was carried out at −80°C overnight. Protein removal was done by centrifugation (10 min at 4°C, 14000 rpm). For subsequent metabolite quantification, the sample supernatant was evaporated to dryness at 40°C in a Speed‐Vac under a gentle nitrogen stream. The samples were then resuspended in 150 μL of water (HPLC grade) with 0.1% formic acid, vortexed for 30 s and centrifuged for 10 min at 4°C, 14000 rpm. For measurements, 75 μL of the supernatant were transferred to a MS vial with inserts (2 mL screw cap brown glass vials, Wicom # WIC41160; inserts f N11‐1 HP, 200 μL, Macherey‐Nagel # 702813; screw caps N9, Macherey‐Nagel # 702732). Specific metabolite calibrators were used to determine sample concentrations. Metabolite quantification was done via HPLC‐MS/MS using a QTrap5500 triple quadrupole mass spectrometer (SCIEX). Depending on the experiment, the LLOQ for each measured metabolite was 0.136–0.428 pmol/sample for TRP, 0.188–0.908 pmol/sample for kynurenic acid, 0.150–0.352 pmol/sample for KYN, 0.060–0.182 pmol/sample for NAD+ and 0.0679–0.399 pmol/sample for 3‐hydroxykinurenine. All targeted metabolomics measurements were carried out by the ZFA Metabolomics unit, Institute of Pharmacology at the Hannover Medical School, Hannover, Germany.

To correct for intersample variations in starting material, the remaining protein pellet after tissue homogenization was dried at room temperature, resuspended in 0.1 M NaOH, vortexed and heated until dissolution (at 95°C for 2 h). The samples were centrifuged (10 min at 4°C, 14000 rpm) and the supernatant was used for protein quantification. The concentration was determined using the Pierce BCA Protein Assay Kit (Thermo Fisher Scientific, Waltham, MA, USA) following the manufacturer's instructions. The absorbance at 540 nm was read with a Tecan Sunrise plate reader (Tecan, Männedorf, Switzerland). The protein concentration was calculated using a BSA standard curve; the average of two replicates for each sample was used in the normalization of the mass spectrometry metabolite quantifications.

### 2.5 Statistical Analysis

All statistical analyses were carried out and plotted using GraphPad Prism software (v 9.5.1 La Jolla, CA, USA). To test if there is a significant association between the treatment (morpholino injection or Kmo inhibition) and whether the zebrafish larvae develop edema (P2 + P3 + P4 from the phenotypic classification described above) or not (corresponding to the larvae categorized as P1), a two‐tailed Fisher's exact test was used. For comparisons between multiple groups, ANOVA was used. When comparing two groups, Student's *t*‐test was used instead. A *p*‐value below 0.05 was considered statistically significant. Other details, such as post hoc tests used and sample sizes, are detailed in each figure legend.

## 3 Results

### 3.1 Morpholino‐Induced KD of the KP Enzymes Leads to Edema Formation and Proteinuria in Zebrafish

**FIGURE 2:**
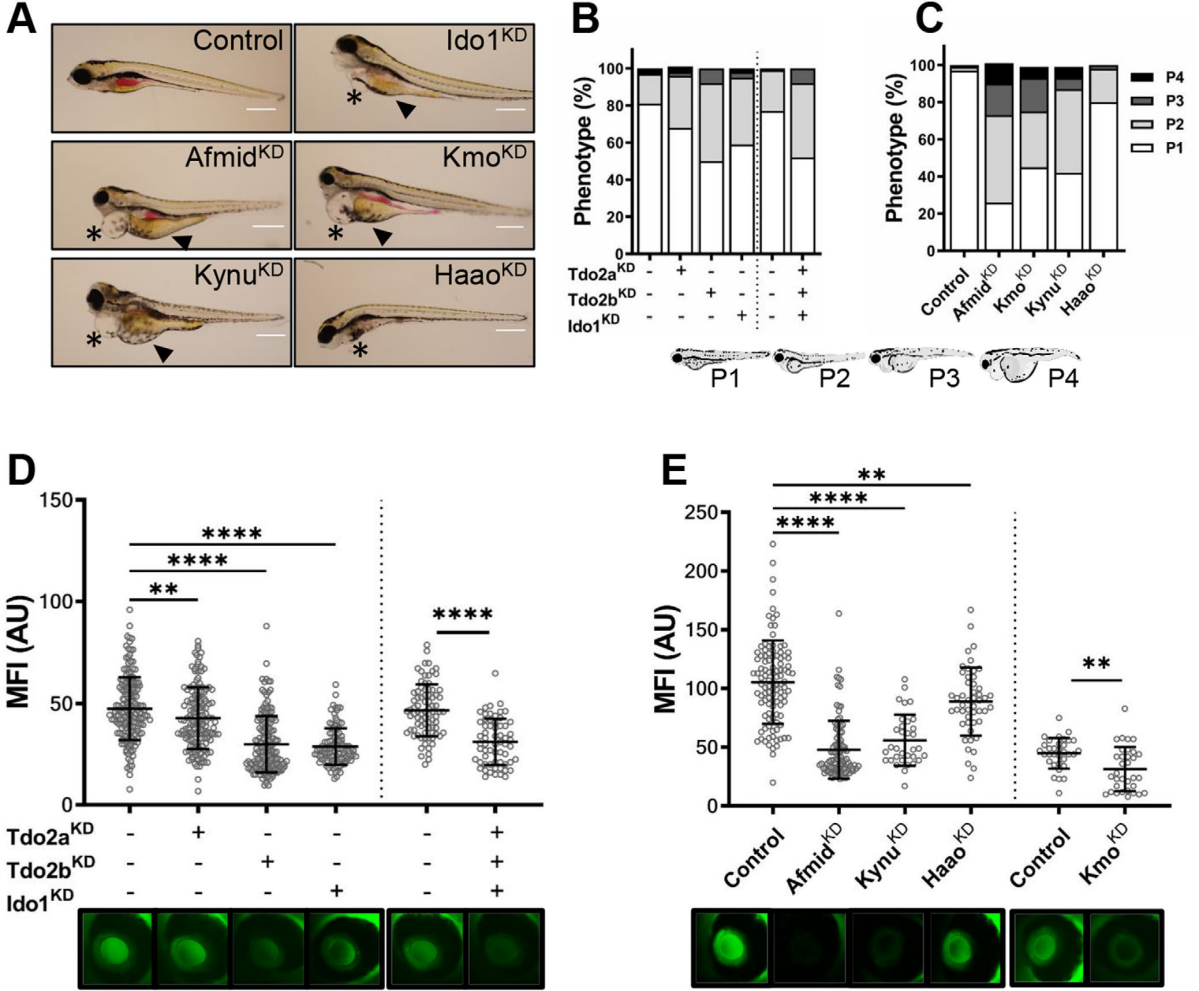
Morpholino‐induced KD of the enzymes of the kynurenine pathway leads to edema formation and proteinuria in zebrafish. Targeted knockdown of the enzymes of the main axis of the kynurenine pathway was carried out by morpholino injection in zebrafish embryos at the 1–4 cell stage. Phenotypic readouts regarding edema formation, ranging from P1 = no edema to P4 = very severe edema, as well as quantification of proteinuria, were recorded at 96 hpf. Proteinuria was assessed by measuring the maximum fluorescence intensity (MFI) of circulating eGFP‐DBP in the retinal vessel plexus of the larvae; a reduction in the signal is used as an indicator of proteins being lost from the circulation. (A) Representative phenotypes of the morphant zebrafish larva at 96 hpf. Knockdown of the enzymes induces yolk sac edema (▲) and pericardial effusion (*) in the injected larvae. Scale bar = 500 μm. (B) Proportion of affected larvae after knockdown of Ido1, Tdo2a and Tdo2b, represented as percentages. (C) Quantification of the larvae belonging to each phenotype category according to the severity of edema formation after disruption of the kynurenine enzymes downstream of the Ido/Tdo node, data shown as percentages. (D) Knockdown of Ido1, Tdo2a and Tdo2b induces proteinuria, as shown by a reduction in the fluorescence signal from the circulating eGFP‐tagged protein. (E) The reduction of the intravascular MFI after knockdown of all other enzymes from the main axis of the pathway shows that their dysregulation also leads to proteinuria. Below each graph showing the MFI quantification is a photograph of the retinal blood vessel plexus of a larva representative for each of the groups (*n* > 30 for all groups; graph represents the mean intensity for each group, each circle shows the maximum fluorescence for one individual fish; error bars are SD; **p ≤ 0.01, ***p ≤ 0.001, ****p ≤ 0.0001; ANOVA and Dunnett's multiple comparison test; when only one group is compared against controls, unpaired *t*‐test was used).

Systemic changes in the catabolic flow of TRP through the KP have been shown to influence a variety of cellular processes; therefore, we examined the effects of morpholino (MO) mediated knockdown of the enzymes of the KP on renal protein excretion and edema formation in zebrafish larvae. After the induction of knockdown by MO injection into 2–4 cell stage embryos, larval development was monitored over the following 96 h. At 96 h postfertilization (hpf), each larva was scored based on the presence and severity of the pericardial effusion and yolk sac edema. The classification ranged from P1 where the larvae appear unaffected to P4 when they show signs of severe edema. Individual knockdown of the three rate‐limiting enzymes of the pathway Tdo2a, Tdo2b and Ido1 leads to the development of edema (Tdo2a *p* = 0.0047; Tdo2b < 0.0001, Ido1 *p* < 0.0001). The increase is more prominent in the moderate (P2) edema category. Tdo2a^KD^ and Ido1^KD^ have a stronger effect on edema formation with a 5.7 and 14.2‐fold increase in severe edema phenotypes (P3–4), respectively. Downregulation of the three enzymes together does not increase the amount of fish with edema when compared to Ido1 alone (*p* = 0.3406. Figure 2A,B). Targeted downregulation of the enzymes Afmid, Kmo and Kynu results in severe generalized edema in over 50% of the cases, with more than a 10‐fold increase in the proportion of severely affected phenotypes (P3–P4) (*p* < 0.0001; *p* = 0.0003; *p* < 0.0001, respectively. Figure 2A,C). To determine if renal protein loss accompanies the development of generalized edema we used a previously established transgenic zebrafish model (Tg(*l‐fabp*:eGFP‐DBP)) that expresses an eGFP‐tagged protein with a size of 78 kDa that, when the glomerular filtration barrier is intact, accumulates in the circulatory system of the larva and can be imaged in the retinal vessel plexus. Once the GFB is damaged, however, proteins are lost from the circulation, evidenced by a reduction in the fluorescence signal [42, 43, 45]. Knockdown of Tdo2b and Ido1 significantly reduced maximum fluorescence by almost 40%, while Tdo2a^KD^ induced only mild proteinuria (10% reduction in MFI). The KD of all three enzymes together also leads to proteinuria, but without an additional increase in protein excretion when compared to the single MO injections (Figure 2D). In line with the severity of the edema formation observed, the loss of proteins from the circulation was greater in the Afmid, Kmo, or Kynu morphant larvae, with a 55%, 30% and 47% maximum fluorescence loss, respectively (Figure 2E).

The KP is the major catabolic route for TRP and it results in the production of a series of bioactive metabolites whose profile is expected to change in response to the targeted reduction in the expression levels of the KP enzymes after MO knockdown. To ascertain if the MO injection leads to metabolite accumulation, we collected morphant zebrafish larvae at 96 hpf for targeted quantification by mass spectrometry. For this analysis, we focused on those enzymes that result in a stronger renal phenotype after knockdown and which are directly involved in regulating systemic KYN abundance, namely Afmid, Kmo and Kynu. Afmid^KD^ leads to a small increase in the amount of TRP and a 35% reduction in the KYN/TRP ratio when compared to controls, suggesting that even when there is a slight increase in the TRP that is available in the Afmid morphant fish, less of it gets processed into KYN (Figure S2A,B). Downstream of Afmid in the TRP catabolism pathway is Kmo, and its activity dictates whether KYN accumulates or gets converted into 3‐hydroxykynurenine (3‐HK). Consistent with a reduction in Kmo expression, the morpholino injected larvae show an 88% reduction in the 3‐HK/KYN ratio, when compared to controls (Figure S2C). Alongside the elevated KYN levels, the Kmo^KD^ larvae also show a trend towards an accumulation of kynurenic acid (KYNA), another metabolite upstream of Kmo (Figure S2D). The enzyme Kynureninase (Kynu) uses both KYN and 3‐HK as substrates to catabolize two separate steps in the KP. Consistent with what would be expected after a downregulation of Kynu expression, our metabolite analysis of Kynu morphant larvae shows an accumulation of 3‐HK, as well as an increase in KYN and KYNA levels (Figure S2E–G).

### 3.2 Chemical Inhibition of Kmo by UPF648 in Zebrafish Larvae Also Induces Edema Formation and Circulatory Protein Loss

**FIGURE 3:**
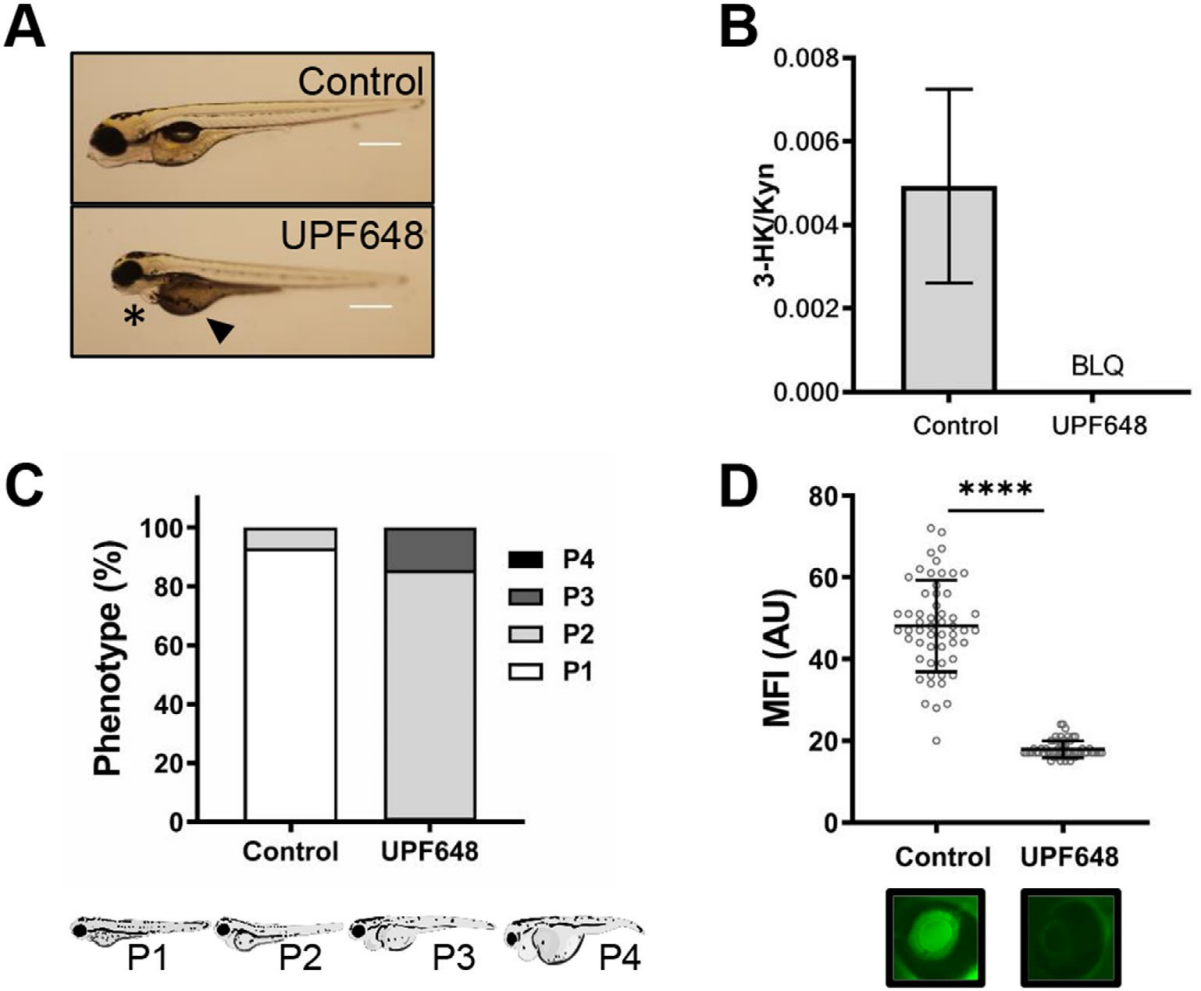
Inhibition of Kmo by UPF648 also leads to the development of hallmarks of proteinuric kidney disease in zebrafish larvae. Zebrafish larvae were treated with the Kmo inhibitor UPF648 at a concentration of 100 μM or with EtOH control after the hatching period (48 hpf) and for the following 48 h. Exposure to the chemicals was done through immersion treatment. Larval development was monitored until 96 hpf and readouts of edema formation and proteinuria were collected. (A) Representative image of the zebrafish larvae after inhibition of Kmo activity by treatment with UPF648. A higher proportion of larvae show signs of pericardial effusion (*) and yolk sac edema (▲) when compared to controls. (B) A reduction in the 3‐HK/KYN ratio indicates a reduced catabolism of kynurenine by Kmo after inhibition with UPF648. All metabolite quantifications have been corrected for variations in protein content in each group, data collected from two independent experiments, each with 10 fish per treatment. BLQ = below level of quantification. (C) Quantification of the proportion of larvae belonging to each of the phenotypic categories based on the severity of the generalized edema. (D) A reduction in maximum fluorescence intensity (MFI) measurement shows that the larvae develop proteinuria upon Kmo inhibition by UPF648. Below the graph denoting the MFI quantification is a representative photograph of the retinal vessel plexus of a larva for each group (*n* > 50 fish for both groups from two independent experiments; horizontal lines represent the mean intensity for each group, each circle shows the maximum fluorescence for one individual fish; ****p ≤ 0.0001; unpaired Student's *t*‐test with Welch's correction).

To validate the phenotype obtained by MO knockdown in our zebrafish model, we chose to inhibit Kmo by treatment with the specific chemical inhibitor UPF648. For this, we administered UPF648 or ethanol (as vehicle control) to the developing larvae via the ERM, at a concentration of 100 μM (total ethanol concentration for both groups was < 0.2% per well). The treatment was started at 48 hpf and continued until 96 hpf when phenotypic readouts for edema and proteinuria were recorded. Strikingly, over 80% of the larvae treated with the Kmo inhibitor developed mild edema (P2), representing more than a 10‐fold increase compared to controls. Additionally, 15% of the treated fish had severe edema, a phenotype not observed in the corresponding controls (*p* < 0.0001, Figure 3A,C). Kmo inhibition also increased circulatory protein loss, as evidenced by a 2.6‐fold reduction in the maximum fluorescence intensity in the retinal vessel plexus of the UPF648 treated larvae when compared to the controls (Figure 3D). Consistent with our previous results from MO knockdown of Kmo, chemical inhibition of the enzyme also results in a marked accumulation of KYN and KYNA, the metabolites upstream of Kmo (Figure S4) and a reduction in the KYN to 3‐HK ratio, which confirms that the KP was successfully interrupted (Figure 3B).

### 3.3 Increasing the KYN Load in Zebrafish Larvae Results in Mild Proteinuria

**FIGURE 4:**
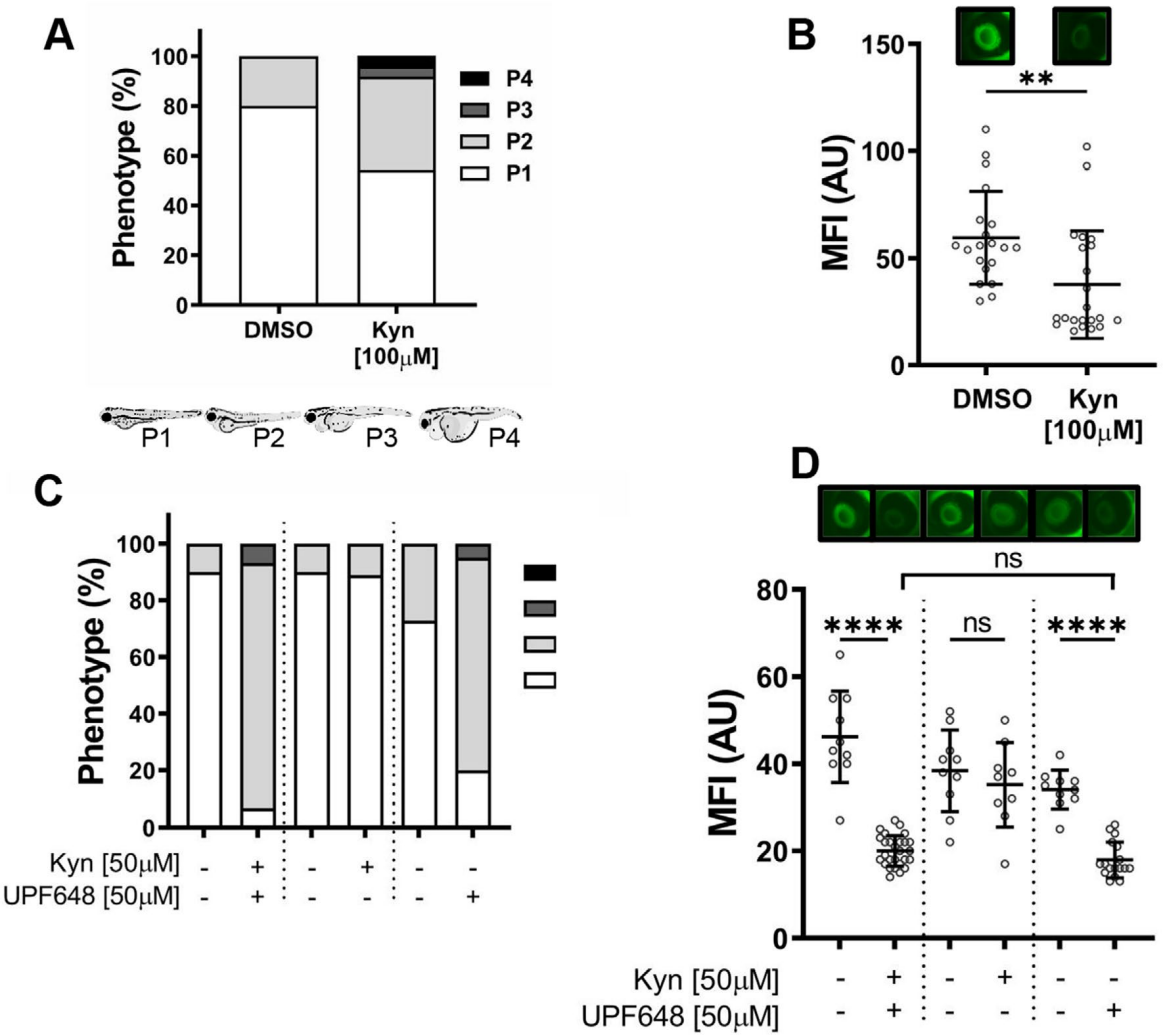
Increasing the kynurenine load in zebrafish larvae results in mild proteinuria and edema formation. Hatched zebrafish larvae were treated either with kynurenine (KYN) or UPF648 at the stated concentrations, starting at 48 hpf, and for the following 48 h. The treatment was delivered via the ERM. Larval development was monitored until 96 hpf; at this time, readouts of edema formation and proteinuria were collected. (A) KYN treatment increases the proportion of larvae that develop mild to severe edema (P2–P4) when compared to the DMSO controls. (B) Quantification of the MFI shows that kynurenine treatment increases renal protein excretion. (C) Percentage of fish in each of the phenotypic categories after treatment with KYN, UPF648, a combination of both, or the respective controls. (D) MFI quantification of the retinal vessel plexus of the larvae after treatment with KYN, UPF648, a combination of both, or the respective controls. Above each MFI graph is a representative photograph of the retinal vessel plexus of a larva from each group (*n* > 10 fish for all groups pooled data from 1 to 2 independent experiments; horizontal lines represent the mean intensity for each group; each circle shows the maximum fluorescence for one individual fish; **p ≤ 0.01, ****p ≤ 0.0001; comparison between two groups was done by unpaired Student *t*‐test with Welch's correction; three or more groups were compared using one‐way ANOVA and Holm–Šídák multiple comparison post hoc test).

Since the manipulation of some of the enzymes of the KYN pathway, particularly Kmo and Kynu, leads to an accumulation of the metabolite KYN, we then assessed if an increase in KYN levels is partly responsible for the renal phenotype observed. For this, we treated the developing larvae with KYN via the standard E3 media for a total of 48 h. At 96 hpf, both edema and proteinuria phenotypes were recorded. When KYN was added to the media at a concentration of 10 μM, the larvae developed normally with a nonsignificant 15% increase in the cases of mild edema (P2; *p* = 0.2591) and there was no evidence for proteinuria (Figure S5). When the concentration was increased to 100 μM, there was more than a 2‐fold increase in the number of edema cases (P2‐P4) of which 37.5% had mild edema (P2) and 8.3% had moderate to severe edema (P3‐P4); however, this increase did not reach statistical significance (*p* = 0.1114, Figure 4A). The increase in edema formation was accompanied by a mild (25%) but significant loss of fluorescent signal from the circulation, which suggests that an increase in KYN could contribute to the onset of proteinuria (Figure 4B). To determine if an increase in KYN influx into the pathway would worsen the phenotype that results from Kmo inhibition, we treated developing zebrafish larvae with either the Kmo inhibitor, KYN or a combination of both via the E3 media. Consistent with what was seen previously, a majority of the larvae treated with the Kmo inhibitor developed generalized edema and proteinuria. When the fish were subjected to an additional KYN load alongside Kmo inhibition, the proportion of fish that developed mild to severe edema increased by 13%, however, this was not statistically significant (*p* = 0.1457 Figure 4C). The intravascular fluorescence intensity showed a trend towards further reduction (11% decrease) when compared with the group that was only treated with the Kmo inhibitor; however, this was also not statistically significant (Figure 4D).

### 3.4 KMO Inhibition Impacts Cell Shape, Size and Focal Adhesions in Cultured Human and Murine Podocytes

**FIGURE 5:**
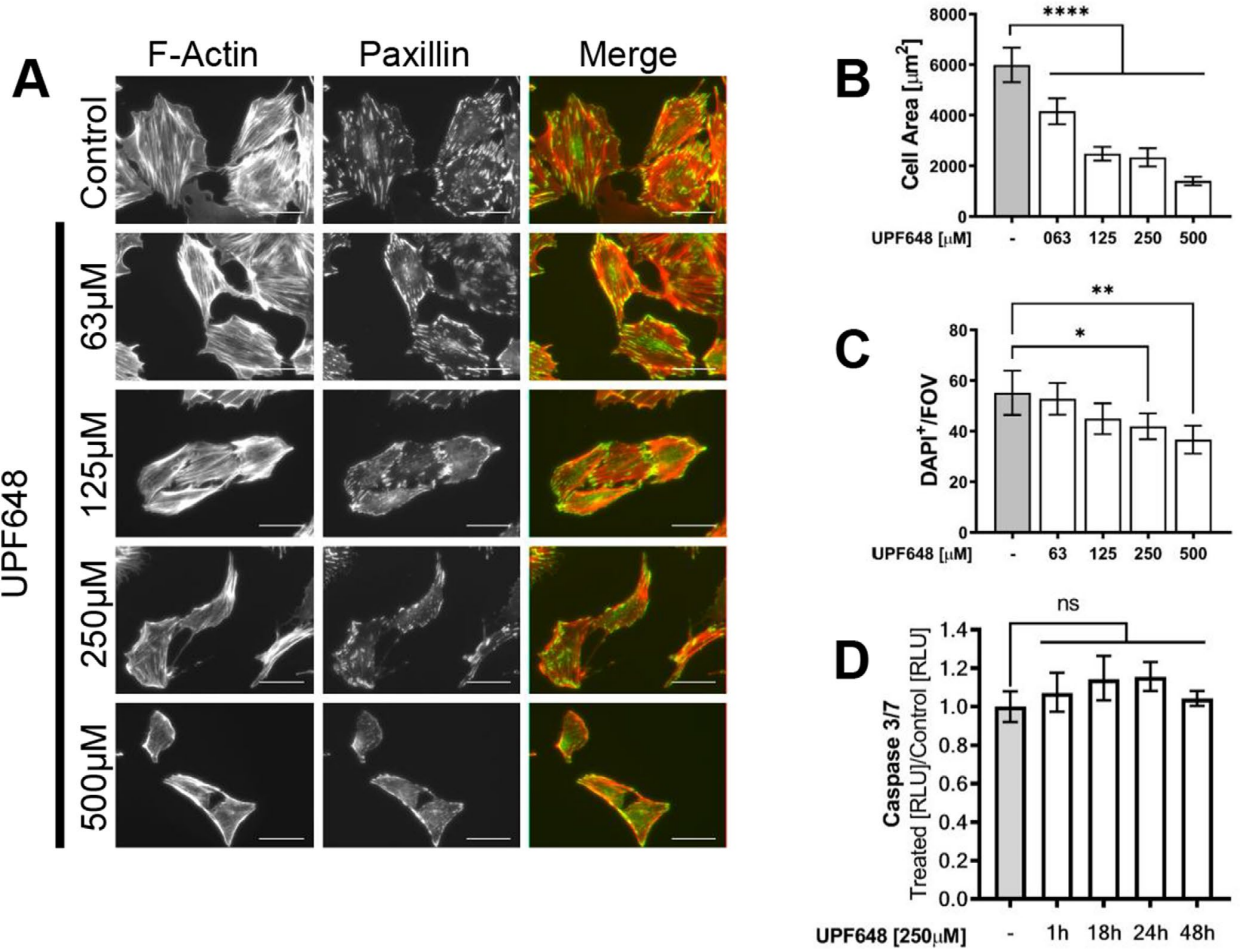
KMO inhibition impacts cell shape, size and focal adhesions in cultured murine podocytes. Mouse podocytes were differentiated on glass cover slides under nonpermissive conditions and then treated with the KMO inhibitor UPF648 or ethanol control at varying concentrations for 48 h. Changes in cell size were assessed by measuring the total cell area in μm^2^ from individual cells across multiple fields of view from two independent experiments. Cell attachment was determined by the number of DAPI^+^ nuclei per field of view (FOV) that are still adherent to the cover slides after KMO inhibition. The cells were photographed at 10X and all nuclei were counted. Thirty photographs were taken for each group, in two independent experiments. (A) Representative images of mouse podocytes after KMO inhibition. Shown are the focal adhesions (paxillin in green) and Actin filaments (phalloidin in red). Photos were taken at 40X. (B) Quantification of changes in the cell size of mouse podocytes after KMO inhibition. Bars represent mean cell area ±95% CI. Photos were taken at 40X. (C) Average number of DAPI‐labeled nuclei from mouse podocytes per FOV. Bars represent the mean cell number per field of view ±95% CI. (D) Apoptosis as measured by Caspase 3/7 activity in mouse podocytes after KMO inhibition, data is presented as a ratio between the luminescence from UPF648‐treated cells relative to the control of the respective time point. Bars indicate the geometric mean of this ratio from at least 3 wells per condition ± geometric SD. Scale bar = 50 μm (**p ≤ 0.01, ***p ≤ 0.001, ****p ≤ 0.0001; ANOVA and Dunnett's multiple comparison test or Kruskal–Wallis test and Dunn's multiple comparison).

**FIGURE 6:**
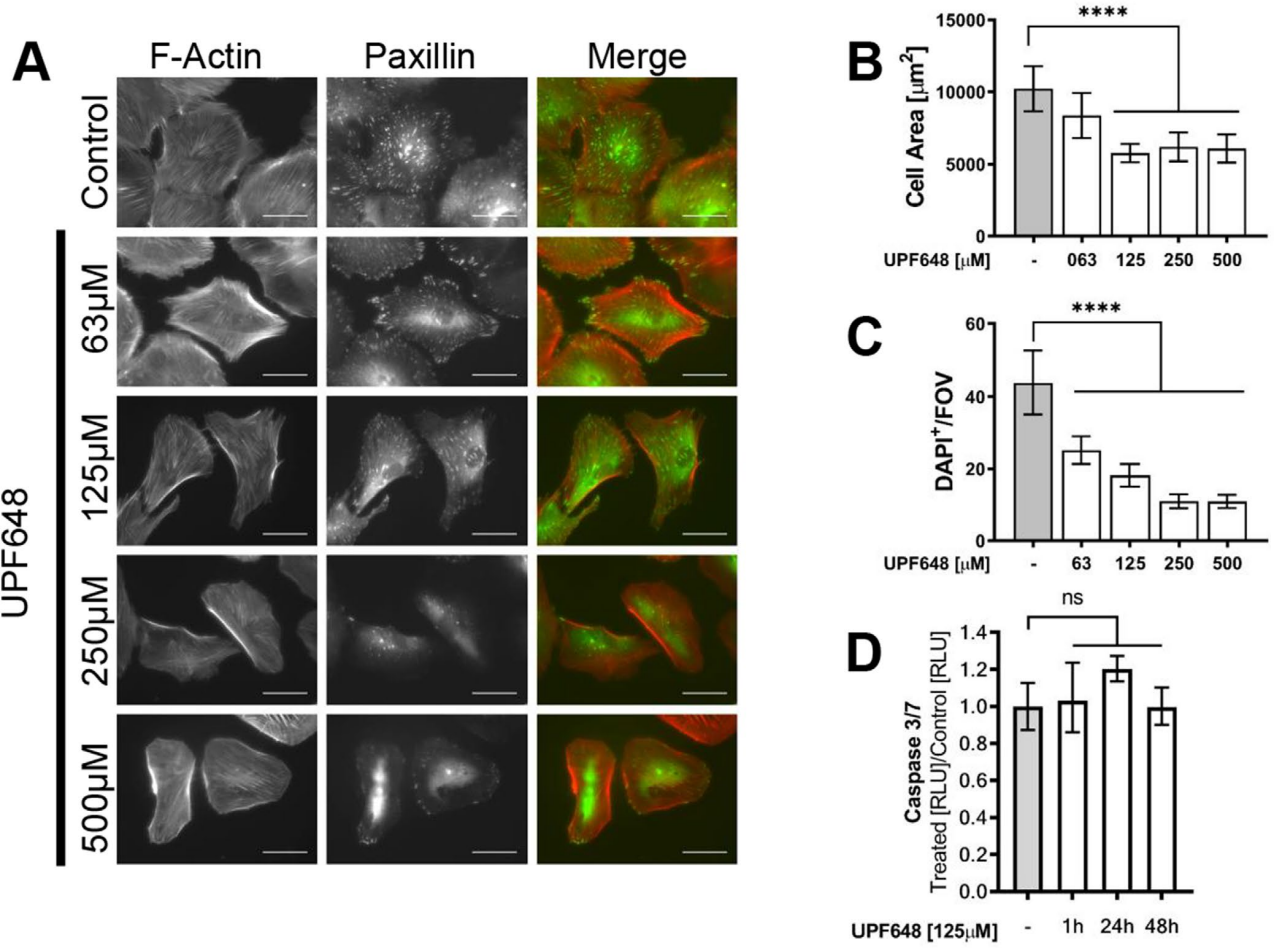
KMO inhibition impacts cell shape, size and focal adhesions in cultured human podocytes. Human podocytes were differentiated on glass cover slides under nonpermissive conditions and then treated with the KMO inhibitor UPF648 or ethanol control at varying concentrations for 48 h. Changes in cell size were assessed by measuring the total cell area in μm^2^ from individual cells across multiple fields of view from two independent experiments. Cell attachment was determined by the number of DAPI^+^ nuclei per field of view (FOV) that are still adherent on the cover slides after KMO inhibition. The cells were photographed at 10X and all nuclei were counted. Thirty photographs were taken for each group, in two independent experiments. (A) Representative images of human podocytes after KMO inhibition. Shown are the focal adhesions (paxillin in green) and Actin filaments (phalloidin in red). Photos were taken at 40X. (B) Quantification of changes in cell size after KMO inhibition in human podocytes. Bars represent mean cell area ±95% CI. (C) Average number of DAPI‐labeled nuclei from human podocytes per FOV. Bars represent the mean cell number per field of view ±95% CI. (D) Apoptosis as measured by Caspase 3/7 activity in human podocytes after KMO inhibition, data is presented as a ratio between the luminescence from UPF648‐treated cells relative to the control of the respective time point. Bars indicate the geometric mean of this ratio from at least 3 wells per condition ± geometric SD. Scale bar = 50 μm (**p ≤ 0.01, ***p ≤ 0.001, ****p ≤ 0.0001; ANOVA and Dunnett's multiple comparison test or Kruskal–Wallis test and Dunn's multiple comparison).

**FIGURE 7:**
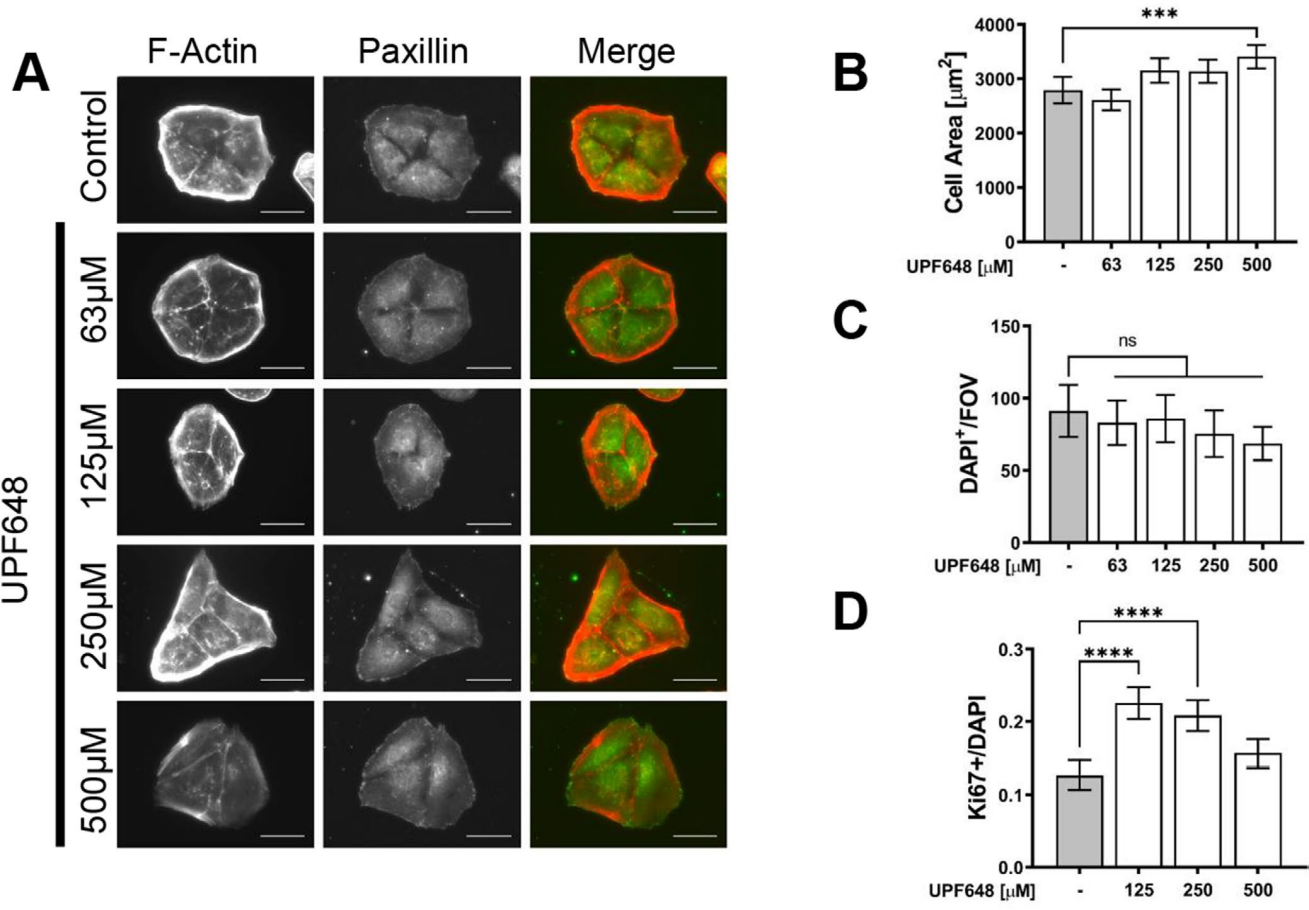
KMO inhibition impacts cell shape, size and proliferation in cultured murine parietal epithelial cells. Mouse parietal epithelial cells (PEC) were seeded on glass cover slides and treated with the KMO inhibitor UPF648 or ethanol control for 48 h at the concentrations stated in the figure. (A) Representative images of mouse PECs after KMO inhibition. The focal adhesions (paxillin in green) and Actin filaments (phalloidin in red) are shown. Scale bar 50 μm. Photos were taken at 40X. (B) Quantification of changes in cell size after KMO inhibition. Total cell area in μm^2^ was quantified from over 80 photographs taken at 40X of phalloidin‐labeled PECs. Bars represent mean cell area ±95% CI. Data from two independent experiments (C) Average number of DAPI^+^ nuclei per field of view (FOV). The cells were photographed at 10X and all nuclei were counted. Twenty photographs were taken for each group, data from two independent experiments. Bars represent the mean cell number per FOV ±95% CI. (**p ≤ 0.01, ***p ≤ 0.001, ****p ≤ 0.0001; ANOVA and Dunnett's multiple comparison test). (D) Nuclei of proliferating cells were stained with Ki‐67, and all nuclei were detected with DAPI. Cells were photographed at 10X and the total DAPI^+^ and Ki‐67^+^ nuclei were counted. Data are presented as a ratio of Ki‐67^+^/DAPI^+^ cells. Bars correspond to the geometric mean per treatment group, error bars indicate 95% CI (40 fields of view were analyzed per treatment, distributed over two independent experiments; ****p ≤ 0.0001; one‐way ANOVA and Dunnett's multiple comparison test).

Since the zebrafish larvae show signs of proteinuric kidney disease after KP dysregulation, we analyzed the effects of KP inhibition on cultured glomerular cells. The focus was placed on KMO because of its role in catabolizing KYN, a metabolite that has been associated with pathological conditions when dysregulated. Consistent with what we have previously shown in vivo [16], both podocytes and parietal epithelial cells express KMO even under in vitro culture conditions. The KMO signal is detected in the cytoplasm of the cells, with a perinuclear distribution and it colocalizes with a mitochondrial marker, as expected from the described expression domain for the enzyme (Figure S6). Since maintaining cell shape and cytoskeletal structure are of vital importance for podocyte function, we analyzed the effects of KMO inhibition on cell morphology upon treatment with UPF648. KMO inhibition leads to changes in cell shape and a reduction in size in both human and mouse podocytes with increasing concentrations of the inhibitor. At the maximum concentration used, mouse podocytes have an 80% reduction in area (Figure 5A,B) and human cells are 50% smaller (Figure 6A,B) when compared to controls. In contrast, mouse parietal epithelial cells (PEC) have a small but significant trend towards an increase in cell area (Figure 7A,B). In addition to the observed morphological changes, podocytes also show alterations in the focal adhesions upon KMO inhibition, as is shown by paxillin staining (Figures 5A,B, and 6A,B; middle column). This could partly explain the significant detachment observed where less than 30% of the human podocytes and 70% of the mouse podocytes remain attached to the surface of the cover slide after UPF648 treatment (Figures 5C and 6C). Since apoptotic cell death could also explain the changes in cell number after treatment, we assayed caspase 3/7 activity after KMO inhibition and were not able to detect a significant increase in cell death at the time points assayed (Figures 5D and 6D). Contrary to what was seen in podocytes, cell surface attachment appears to be unaltered by UPF648 in PECs (Figure 7C) and there is an increase in the ratio of Ki‐67^+^ cells after UPF648 treatment (Figure 7D), suggesting that an increase in proliferative activity could come as a consequence of KMO inhibition in PECs.

### 3.5 There Are Changes in Cellular Energy and Redox Homeostasis in Cultured Podocytes as a Result of KMO Inhibition

**FIGURE 8:**
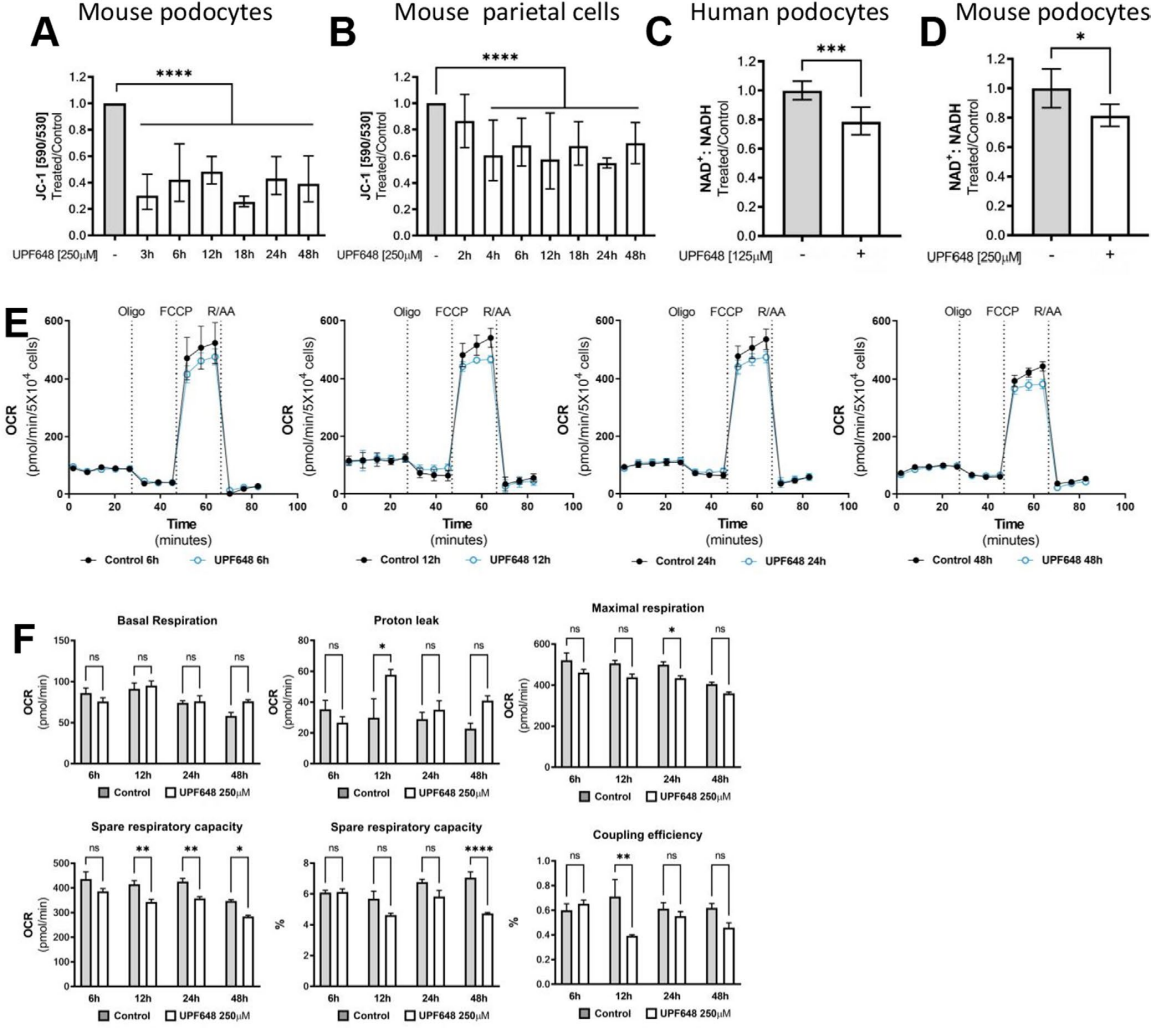
Changes in cellular energy and redox homeostasis in cultured podocytes after KMO inhibition. Murine glomerular cells were treated with the KMO inhibitor UPF648 and different aspects of mitochondrial health and bioenergetic parameters were assessed. Changes in the spectral profile of the mitochondrial dye JC‐1 show that upon KMO inhibition, both (A) podocytes and (B) parietal cells have an increase in mitochondrial membrane depolarization. Data is presented as the ratio between 590/530 nm relative to the control for each of the time points shown. Bars represent the geometric mean ± geometric SD from 3 to 13 wells pooled from 3 to 4 independent experiments for each cell type (**p ≤ 0.01, ****p ≤ 0.0001; groups were compared using one‐way ANOVA and Holm–Šídák multiple comparison post hoc test). The redox state of the NAD cofactor was assessed using a plate‐based assay that allows for the separate quantification of NAD+ and NADH. For this, the podocytes were allowed to differentiate under nonpermissive conditions and then treated with UPF648. Both human (C) and mouse (D) podocytes show a significant reduction in the NAD+/NADH ratio as a consequence of KMO inhibition. Bars indicate the geometric mean of values from two independent experiments ± geometric SD expressed as a fold‐change relative to controls (*p < 0.05, ***p ≤ 0.001; unpaired *t*‐test). (E) Oxygen consumption rate (OCR) traces and (F) summary of mitochondrial bioenergetic parameters extracted from the seahorse traces for each time point of KMO inhibition by UPF648 in adherent mouse podocytes. Data presented as the average of 4–5 wells per time point ± SEM (n.s. = nonsignificant, *p < 0.05, **p ≤ 0.01, ***p ≤ 0.001, ****p ≤ 0.0001; one‐way ANOVA and Holm–Šídák multiple comparison post hoc test). FCCP, Carbonyl cyanide‐4 (trifluoromethoxy) phenylhydrazone; Oligo, Oligomycin; R/AA, Rotenone and Antimycin A.

As KMO is located in the mitochondria and the KP results in the de novo synthesis of NAD, we then assessed if the inhibition of KMO by UPF648 would result in an impact on mitochondrial health and energy metabolism in cultured glomerular cells. As an initial approach, we used the JC‐1 dye, which shifts its emission spectrum from 590 ± 35 nm for its aggregate form in mitochondria with high membrane potential to 528 ± 20 nm in its monomeric form in depolarized mitochondria. Treatment with the KMO inhibitor leads to a significant depolarization of the mitochondria in both mouse podocytes and parietal epithelial cells. Notably, once again the podocytes appear to be more sensitive to UPF648 treatment showing a strong depolarization of more than 50% when compared to control‐treated cells, staying below this value for all of the time points included in this experiment (Figure 8A and Figure S7). PECs experience a gradual loss of mitochondrial membrane potential that only reaches significance after 4 h of treatment and is overall milder than that seen in the podocytes (Figure 8B). As mentioned before, these changes in mitochondrial membrane potential do not seem to correlate with an increase in cell apoptosis, as measured by the caspase assay (Figures 5D and 6D) for the time points analyzed.

To determine if inhibition of the KP would have an impact on NAD availability for the cultured cells, we measured total NAD levels via mass spectrometry after treatment with UPF648. In these experiments, human podocytes appear to be more susceptible to disruptions in the de novo synthesis pathway for NAD, with a 55% reduction in total NAD after 48 h in the presence of the KMO inhibitor (Figure S8A). The mouse cells, on the other hand, showed a different pattern, with an increase in NAD content after 48 h in the presence of the inhibitor (Figure S8B–C). However, when shorter incubation periods were analyzed, both mouse glomerular cell types, but especially the podocytes, seem to have an initial reduction in total NAD, possibly followed by compensatory changes that alter the availability of NAD in later time points (Figure S8E–F). When we proceeded to analyze if there were changes in the redox state of NAD in the podocytes in response to KMO inhibition, both human and mouse podocytes showed a small but significant reduction in the ratio of NAD+ to NADH when compared to the controls without KMO inhibition (Figure 8C,D).

In light of these results, we proceeded to analyze the bioenergetic parameters related to mitochondrial stress in cultured murine podocytes upon KMO inhibition, using a microplate‐based assay for extracellular flux analysis (Seahorse XF Mito Stress Test, Agilent). Bioenergetic readouts in mouse podocytes were carried out at different time points between 6 and 48 h from the start of the KMO inhibition. No differences were observed in the basal respiratory rate in the examined time points; however, the oxygen consumption rate (OCR) related to maximal respiration and spare respiratory capacity was reduced in the cells that were subjected to the inhibition of the KYN pathway, In the case of the spare respiratory capacity, this change becomes significant after 12 h of inhibition and remains so until the end of the experiment. Additionally, a trend towards increased proton leak and decreased coupling efficiency was also seen upon KMO inhibition (Figure 8F,G).

These results derived from the in vitro work with the glomerular cells suggest that podocytes are generally more susceptible to damage due to KYN dysregulation, particularly when it comes to changes that negatively impact the cytoskeleton and energy homeostasis. Taken together with the in vivo results, we show that systemic alterations in KYN metabolism as a consequence of changes in enzyme expression or activity may contribute to a functional impairment of the glomerular filtration barrier, ultimately leading to the excretion of proteins from the circulation.

## 4 Discussion

The systemic and cellular availability of TRP is determined by a balance between dietary input and the multiple pathways involved in its catabolism. The KP accounts for the majority of TRP degradation, and since both excess and depletion of TRP have been deemed detrimental to health, precise but flexible regulation of the pathway is vital [1]. Although the liver is responsible for the largest proportion of TRP catabolism, the kidneys also express the entire enzymatic machinery needed for the KP and they are the only route for the excretion of the KYN metabolites [28]. Therefore, the impact of dysregulated TRP catabolism can come from the endogenous metabolites generated by the kidney cells, as well as the KYNs produced systemically that diffuse into the bloodstream and reach the filtration barrier.

In 1983, Kaysen and Kropp showed indirect evidence linking changes in circulating TRP levels and the development of proteinuria, using an experimental model of uremia in rats. In their experiments, they were able to establish that TRP supplementation prevents the onset of proteinuria and hypertension but is not sufficient to avoid the histological changes that characterize the progression to end‐stage renal disease [49]. On the other hand, increased TRP oxidation has been associated with systemic complications that result from kidney disease. For example, KYN metabolite levels were shown to correlate with increasing concentrations of circulating adhesion molecules, oxidative status, endothelial dysfunction and an increased inflammatory state in patients [19]. Specifically, KYN and kynurenic acid have been consistently and robustly correlated with disease progression in patients with renal insufficiency undergoing hemodialysis [21, 24], with the progression to end‐stage renal disease (ESRD) in cases of type 2 diabetes associated CKD [25], with biopsy‐confirmed kidney allograft rejection after transplantation [26, 27], and in animal models of experimental renal failure [28, 29]. All of this body of work is congruent with our observations from the in vivo experiments carried out in the zebrafish larvae where we showed that by disrupting the expression level of the enzymes of the KP by morpholino injections we induce systemic alterations in the TRP metabolites which, in turn, lead to the development of edema and large molecular weight protein loss from the circulation. In the past, our lab has used both of these readouts as consistent and reliable markers across multiple studies to inform the status of the glomerular filtration barrier in the zebrafish larvae [15, 43, 45, 50, 51, 52, 53, 54].

IDO and TDO are two structurally distinct but functionally convergent enzymes that catalyze the first step of the KYN pathway, resulting in the production of N‐formylkynurenine [55]. In zebrafish, two tdo paralogues resulted from a gene duplication event (tdo2a and tdo2b), and the one ido gene sequence more closely resembles that of Ido2 in mammals; therefore, there are three enzymes capable of carrying out the first catalytic step in TRP degradation [56, 57]. In our experiments, we show that the knockdown of either Ido or Tdo2b, on their own, is sufficient to induce yolk sac edema, pericardial effusion and circulatory protein loss. Tdo2a had only a mild effect on the renal readouts analyzed. These enzymes may have a nonoverlapping expression pattern, and functional divergence of the duplicated gene could explain the lack of compensatory effect of the other enzymes in the individual knockdowns. Not much is known about the impact of Ido and Tdo dysregulation in zebrafish; however, in other vertebrate models, others have shown that renal IDO expression exacerbates kidney injury in ischemia–reperfusion by inducing tubular cell apoptosis via the Fas/FasL pathway in response to an inflammatory signal [58, 59]. In contrast to the results described in the injured renal tubules in mice, a study by Anquetil et al. showed that in humans, a reduction in IDO1 expression preceded pancreatic ß‐cell death in type I diabetes [60], suggesting that the physiological role of KYN metabolites and intrinsic signaling pathways mediated by the KP is highly dependent on the cellular microenvironment.

The next step in TRP degradation is catalyzed by AFMID, in which N′‐formylkynurenine is hydrolyzed to produce KYN. In Afmid^KD^ fish, there are also signs of increased protein excretion and development of edema, suggesting that the glomerular filtration barrier could be affected. Additional evidence of renal dysfunction in the context of Afmid deficiency comes from studies carried out by Dobrovolsky et al. in which the removal of the Afmid/Tk locus leads to glomerular sclerosis that results in lethal kidney failure [61]. It is still unclear whether the renal phenotype of Afmid reduction is mediated by direct effects of NFK accumulation or by other signaling pathways related to KYN metabolite availability. Even in the Afmid/Tk double knockout model, the kidneys still retain around 13% of their NFK hydrolyzing activity, suggesting that other non‐NFK specific formamidase enzymes compensate for the loss of Afmid to eliminate NFK and it is possible that this process could be toxic to some cells in the glomerulus [14].

We and others have previously documented the role played by KMO in maintaining kidney function. By QTL mapping, Zhang et al. reported that the gene coding for KMO is located within a genomic region associated with elevated urinary albumin to creatinine ratio [16]. A follow‐up study from our group showed that KMO knockout mice develop albuminuria and partial podocyte foot process effacement, and Kmo knockdown in zebrafish leads to the characteristic phenotypic hallmarks of proteinuric kidney disease [15]. In line with these results, we now show that the Kmo knockdown or enzymatic inhibition by UPF648 reliably and reproducibly led to increased protein excretion and edema formation in zebrafish larvae. Consistent with an interruption of the pathway, we also show that the intervention results in the accumulation of KYN and kynurenic acid, the metabolites upstream of Kmo. This metabolite profile is in line with other reports of KMO inhibition or genetic deletion in rodents [15, 62, 63], where an increase in KYN results in the activation of Kat enzymes, increasing kynurenic acid. However, Zheng et al. showed that kidney damage is attenuated in the absence of KMO during ischemia–reperfusion injury as its absence seems to be protective against tubular necrosis and apoptosis, as well as neutrophil infiltration in mice [12], suggesting that there could be cell‐specific responses.

Once KYN is produced, it can be catabolized by either one of three enzymes: KATs into kynurenic acid, by KMO into 3‐HK and by KYNU into anthranilic acid. Which of these pathways is preferred depends mostly on the cell type, the enzymatic activity and substrate availability, and there appear to be large variations between species [64]. In our morphant fish, we see that upon Kynu^KD^, there is a 10‐fold increase in the amount of 3‐HK, consistent with a reduced activity of Kynu downstream of Kmo. Increased levels of 3‐HK have been previously associated with ROS‐mediated cell death in neurodegenerative disorders [65]. Additionally, 3‐HK has been shown to induce mitochondrial dysfunction that could lead to impaired energy supply and, ultimately, cell death [66]. When it comes to KYN metabolite exposure, Majewski et al. 2018 [67] showed that zebrafish larvae also develop edema and show signs of altered heartbeat frequency after environmental exposure to toxic levels of some of the KYNs. One caveat of our study is that although we monitor embryonic development to ensure the correct formation of a circulatory system, our analysis does not consider how changes in cardiac function may contribute to the phenotypes observed. However, by using both edema and circulatory protein loss as readouts for our experiments, we can show that systemically altering the KP leads to a dysfunction of the glomerular filtration barrier.

The majority of the studies that analyze the consequences of KYN dysregulation typically look at the effects of systemic changes on a specific health outcome, but the complex network of interconnected reactions that use the KYNs as either substrate or as part of a signaling pathway makes it difficult to predict the cellular consequences of said dysregulation. Since our zebrafish experiments altering the KP suggest that the glomerular filtration barrier could be injured, we focused on the impact of KP dysregulation on isolated glomerular cells. Podocytes are highly specialized cells whose function is largely dependent on morphological and structural characteristics supported by a dynamic cytoskeletal network (reviewed in [68]). Our results show that inhibiting KMO leads to alterations in podocyte morphology. The reduction in cell size is also accompanied by a decrease in paxillin staining at the focal adhesions, which could partly contribute to the reduced cell attachment observed in the treated cells. Typically, cells lacking Paxillin have been shown to have altered cell adhesion, abnormal cortical cytoskeletal structure and reduced phosphorylation of important adhesion molecules [69]. The significance of maintaining focal adhesion architecture and density in podocytes is highlighted by the fact that disease‐causing mutations have been identified in different components of the cell attachment system [70, 71] and therapeutic targeting of the podocyte focal adhesions has been proposed in the treatment of proteinuric kidney diseases [51, 72, 73]. Which mechanism is impacted by the KYN metabolites that could lead to cytoskeleton alterations in the podocytes is still an open question; however, it has been shown that in brain cells, both QUIN [74, 75] and KYNA [76, 77] can act as signaling molecules through glutamate and possibly cholinergic receptors. Podocytes have also been shown to possess the functional molecular machinery for glutamate signaling [78] and antagonism of NMDA receptors in podocytes induces profound actin remodeling that leads to increased albuminuria [79]. On a cellular level, changes in glutamate signaling are highly pleiotropic and have been shown to lead to alterations in calcium currents and phosphorylation cascades that affect known cytoskeleton regulators such as CaMKII and Cofilin [79, 80, 81, 82].

The role played by KMO in TRP catabolism is well established; however, it is unclear if its location at the outer mitochondrial membrane is indicative of other cellular functions or if the canonical TRP degradation pathway plays any role in modulating cellular bioenergetics other than being the de novo source of NAD. There is some evidence of tissue‐specific alterations in cellular respiratory parameters in brain, liver and heart mitochondria in response to varying concentrations of extracellular KYN metabolites [83, 84], however, not much is known about the effect on the kidney. Our cell‐based experiments show that in cultured podocytes, KMO inhibition leads to a loss in mitochondrial membrane potential, alterations in the NAD^+^/NADH ratio suggestive of redox imbalances, overall mitochondrial dysfunction and a reduced ability to adjust the metabolic rate in response to stress. Reduced oxygen consumption after the addition of FCCP was consistently seen across different experiments upon KMO inhibition. This indicates that the spare respiratory capacity in the cultured podocytes is impaired. Although we do observe some changes in the bioenergetics parameters of the cultured mouse podocytes, these changes are less striking than the mitochondrial depolarization observed after UPF648 treatment. One possible explanation is that the mitochondrial membrane potential can be reduced substantially while oxidative phosphorylation remains relatively preserved until a threshold is reached [85]. Additionally, there are other examples where mitochondrial membrane depolarization does not have a direct correlation with OCR, which indicates that in some cases mitochondria can, at least temporarily, maintain energy production despite changes in mitochondrial status, providing some level of protection to potentially lethal cellular stressors [86]. Changes in the bioenergetic reserve capacity of a cell are complex and multifactorial and several reasons have been suggested for alterations in said parameter; for example, reduced mitochondrial mass, damages in the electron transport chain, a decrease in substrate availability [87], as well as being reflective of the capacity of podocytes to switch from one source of energy to another to maintain homeostasis [88].

Mitochondrial dysfunction has been associated with podocyte injury in cases of congenital nephrotic syndrome [89], models of FSGS [90], aldosterone‐induced podocyte damage [91, 92] and in cultured podocytes in a diabetic context [93, 94] (also reviewed in [95, 96]). Moreover, the morphological alterations we observed in the podocytes could also be interconnected with the mitochondrial phenotype seen, where ROS production and energetic imbalances due to decreased ATP production have also been associated with general podocyte injury reflected in changes in the cytoskeleton and loss of renal function via multiple pathways [97, 98].

Taken together, our results show that altering TRP catabolism by interfering with the expression of the enzymes at different levels of the pathway leads to the development of edema and proteinuria in zebrafish, suggesting the presence of glomerular damage. Our in vitro data support the notion that KYN dysregulation by KMO inhibition is detrimental to podocytes and results in changes in cell shape, size, substrate adhesion and mitochondrial function. Our study's strength lies in the fact that by using the zebrafish model, we can analyze the effects of KP interference in parallel under nearly identical experimental conditions in a rapidly developing model where the organism depends on a single glomerulus. However, the complexity of the pathway and its scope of influence, together with the limitations of our particular experimental model, do not allow for the assessment of the long‐term effects of chronic exposure to dysregulated KYNs, which would presumably more closely resemble the panorama in humans. Since some mouse models with defects in Kmo or Kynu show no evidence of a progressive renal phenotype leading to kidney disease [15, 99], it suggests that the more complex adult mammalian kidney has several possibilities to compensate for a single enzyme defect. Therefore, it is a possibility that changes in TRP metabolite levels observed in the blood of patients with reduced eGFR could indicate that alterations in TRP metabolism cannot be compensated for in advanced kidney disease states and this could represent a cofactor for the progression of CKD.

## Author Contributions

Mario Schiffer, Hermann Haller, Patricia Bolanos‐Palmieri conceived and planned the experiments. The zebrafish experiments were completed by Patricia Schroder, Lynne Staggs, Heiko Schenk and Patricia Bolanos‐Palmieri. The cell culture experiments were carried out by Patricia Bolanos‐Palmieri. Data analysis, interpretation and manuscript preparation were done by Patricia Bolanos‐Palmieri with the support of Mario Schiffer. All authors provided critical feedback that contributed to the final version of the manuscript.

## Conflicts of Interest

The authors declare no conflicts of interest.

## Supporting information


**Figure S1:** Diagram of a typical oxygen consumption trace measured using a Seahorse Extracellular Flux Analyzer. Explanatory trace diagram based on the information provided by Agilent for the mitochondrial stress test showing the oxygen consumption rate over time, in response to different modulators of the respiratory chain. The shaded areas highlight the bioenergetic parameters that can be calculated.

**Figure S2:** Knockdown of the kynurenine enzymes by morpholino injection results in alterations in the metabolite patterns in the morphant larvae. Zebrafish embryos were injected either with specific morpholinos targeting enzymes of the kynurenine pathway or a scrambled control. The larvae were collected at 120hpf for metabolite analysis by mass spectrometry. (A) Total Trp and (B) Kyn/Trp ratio after Afmid^KD^. (C) Metabolite quantification after Kmo^KD^. A reduction in the 3‐HK/KYN ratio indicates a reduced catabolism of kynurenine by Kmo. (D) Kmo downregulation reroutes the pathway to the production of KYNA, leading to its accumulation in the morphant larva. (E‐G) Knockdown of Kynu results in the accumulation of upstream metabolites KYN, KYNA and 3‐HK. Each sample includes *n* > 10 larvae and each value has been normalized to total protein content. BLQ = Bellow level of quantification.

**Figure S3:** Evidence of knockdown of the kynurenine enzymes. Zebrafish larvae were injected with MO against the enzymes of the kynurenine pathway. At 120 hpf the larvae were lysed and mRNA was used to quantify active transcription of the targeted enzymes: (A) Ido1 (B) Afmid. Data is normalized to Hprt and presented as a ratio relative to control.

**Figure S4:** Kmo inhibition by UPF648 leads to an accumulation of the upstream metabolites. Zebrafish larvae were treated with UPF648 or EtOH control during the hatching period (48 hpf) and for the following 48 h, the compounds were administered via the ERM and the larval development was monitored up until 96 hpf. Quantification of the metabolites upstream of Kmo: (A) kynurenine and (B) kynurenic acid after Kmo inhibition. BLQ = Below level of quantification.

**Figure S5:** An additional low dose of kynurenine is not sufficient to induce a renal phenotype in zebrafish larvae. Hatched zebrafish larvae were treated either with kynurenine at a concentration of 10 μM or with DMSO starting at 48 hpf, and for the following 48 h. The treatment was delivered via the embryo rearing media (ERM). Larval development was monitored until 96 hpf, at this time, readouts of edema formation and proteinuria were collected. (A) Kynurenine treatment at this concentration does not increase the proportion of larvae that develop mild to severe edema (P2‐P4). (B) Additionally, quantification of the MFI shows that kynurenine treatment at 10 μM does not lead to proteinuria (*n* > 10 fish for all groups; bars represent the mean intensity for each group, each circle shows the maximum fluorescence for one individual fish; comparison between two groups was done by unpaired Student *t*‐test with Welch's correction, n.s., nonsignificant).

**Figure S6:** KMO is expressed in cultured glomerular cells and it colocalizes with mitochondria. Human (A) and mouse (B) Immortalized podocytes (POD), as well as (C) murine parietal epithelial cells (PEC) express the enzyme KMO under cell culture conditions. Cells were seeded on glass cover slides and stained with an anti‐KMO antibody (green). Mitochondria were labeled using a MitoTracker fluorescent probe (red). The nuclei are visualized using DAPI (blue). The merged image shows a perinuclear expression pattern and colocalization of KMO with mitochondria, as expected. (D) Western blot of podocyte and parietal epithelial cell lysates shows KMO as a band at the expected size of 55 kDa.

**Figure S7:** KMO inhibition leads to mitochondrial depolarization. Mouse podocytes were treated with the KMO inhibitor UPF648. JC‐1 dye was used to visualize changes in mitochondrial membrane polarization status, showing an increase in mitochondrial depolarization. Representative images taken at 20X show single channels at 530 nm (green) and 590 nm (red), as well as merged (last column). FCCP was used as a positive control to show loss of mitochondrial membrane potential.

**Figure S8:** KMO inhibition shows a difference in total NAD content in response to UPF648 treatment. Glomerular cells were treated with UPF648 at the indicated concentrations and for the specified time points. Cells were lysed after 6, 12, 24 and 48 h of KMO inhibition and were prepared for total NAD quantification by mass spectrometry. NAD quantification after treatment with UPF648 for 48 h (A) human podocytes, (B) mouse podocytes, (C) mouse parietal epithelial cells. NAD quantification after a time course of treatment with UPF648 (D) human podocytes, (E) mouse podocytes, (F) mouse parietal epithelial cells. Values were normalized by total protein content in each sample. Bars indicate the geometric mean of this ratio ± geometric SD, values represent data from 2 independent samples per time point, presented as relative to control.

## Data Availability

The data that support the findings of this study are available in the Materials and Methods, Results, and/or Supporting Information of this article.
