# Supplementary figures and images for: Kynurenine Pathway Dysregulation Impairs Podocyte Morphology and Bioenergetics In Vitro and Leads to Glomerular Dysfunction
Source: FASEB J. 2025 Nov 12;39(22):e71228. doi: 10.1096/fj.202502175R (PMC12611232; doi:10.1096/fj.202502175R)

**Figure S1**

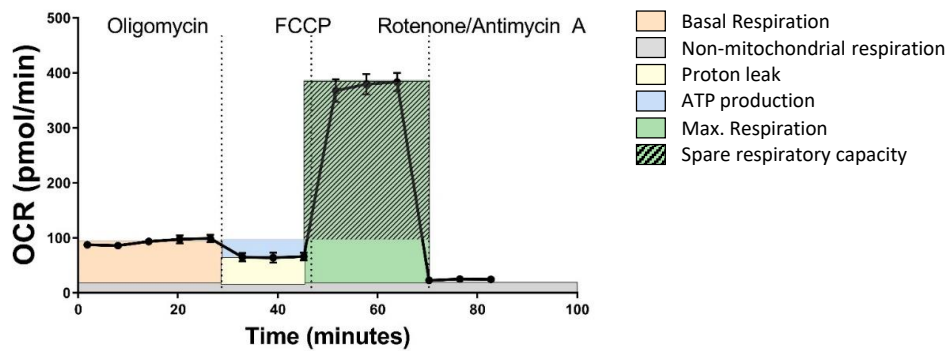

Supplement: Supplementary file 1 — Figure S1: Diagram of a typical oxygen consumption trace measured using a Seahorse Extracellular Flux Analyzer. Explanatory trace diagram based on the information provided by Agilent for the mitochondrial stress test showing the oxygen consumption rate over time, in response to different modulators of the respiratory chain. The shaded areas highlight the bioenergetic parameters that can be calculated. [file FSB2-39-e71228-s007.pdf]

**Figure S2**

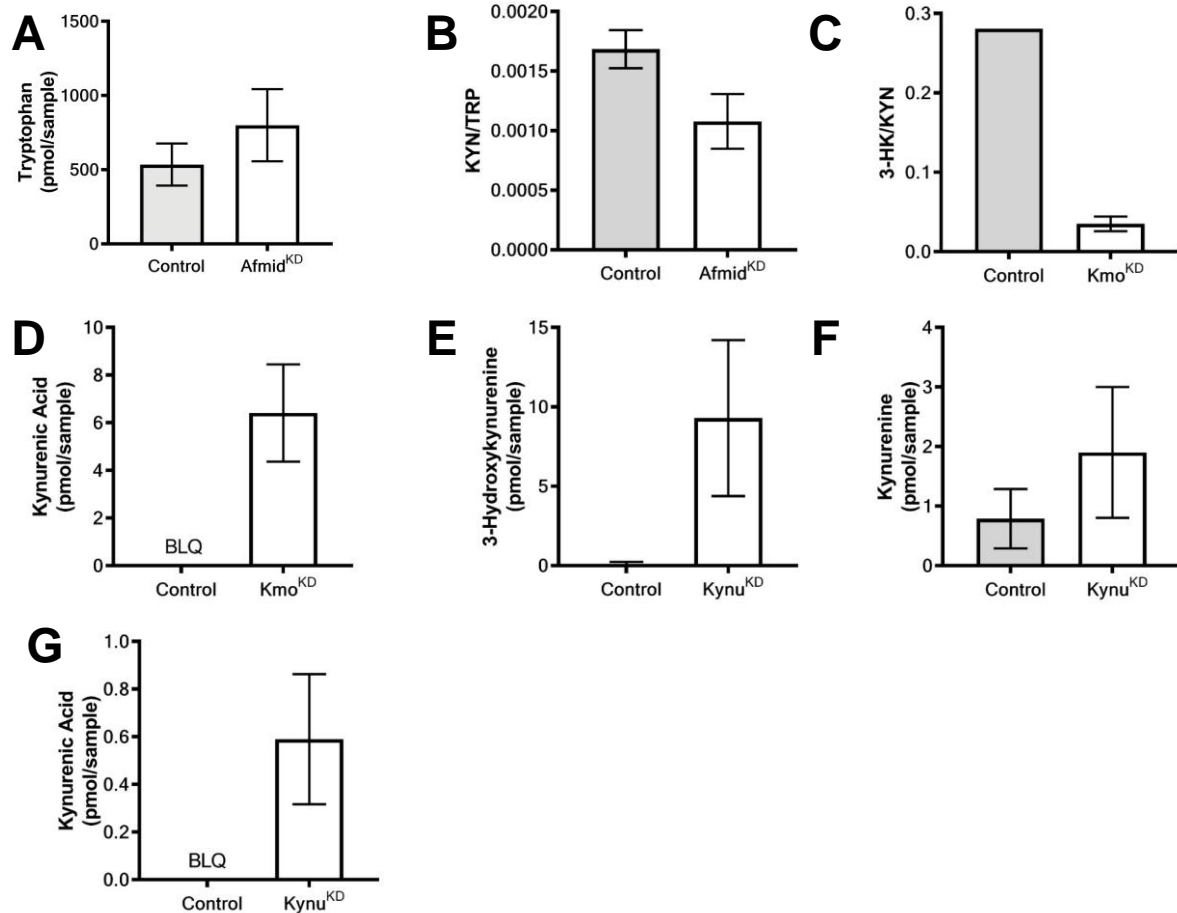

Supplement: Supplementary file 2 — Figure S2: Knockdown of the kynurenine enzymes by morpholino injection results in alterations in the metabolite patterns in the morphant larvae. Zebrafish embryos were injected either with specific morpholinos targeting enzymes of the kynurenine pathway or a scrambled control. The larvae were collected at 120hpf for metabolite analysis by mass spectrometry. (A) Total Trp and (B) Kyn/Trp ratio after AfmidKD. (C) Metabolite quantification after KmoKD. A reduction in the 3‐HK/KYN ratio indicates a reduced catabolism of kynurenine by Kmo. (D) Kmo downregulation reroutes the pathway to the production of KYNA, leading to its accumulation in the morphant larva. (E‐G) Knockdown of Kynu results in the accumulation of upstream metabolites KYN, KYNA and 3‐HK. Each sample includes n > 10 larvae and each value has been normalized to total protein content. BLQ = Bellow level of quantification. [file FSB2-39-e71228-s005.pdf]

Figure S3

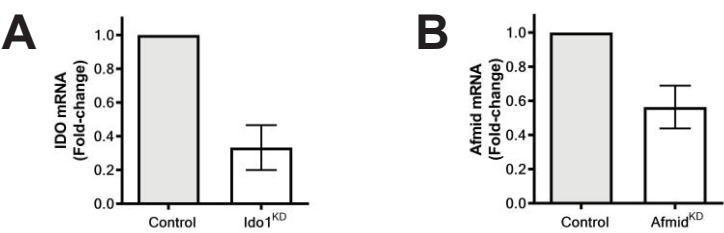

Supplement: Supplementary file 3 — Figure S3: Evidence of knockdown of the kynurenine enzymes. Zebrafish larvae were injected with MO against the enzymes of the kynurenine pathway. At 120 hpf the larvae were lysed and mRNA was used to quantify active transcription of the targeted enzymes: (A) Ido1 (B) Afmid. Data is normalized to Hprt and presented as a ratio relative to control. [file FSB2-39-e71228-s003.pdf]

Figure S4

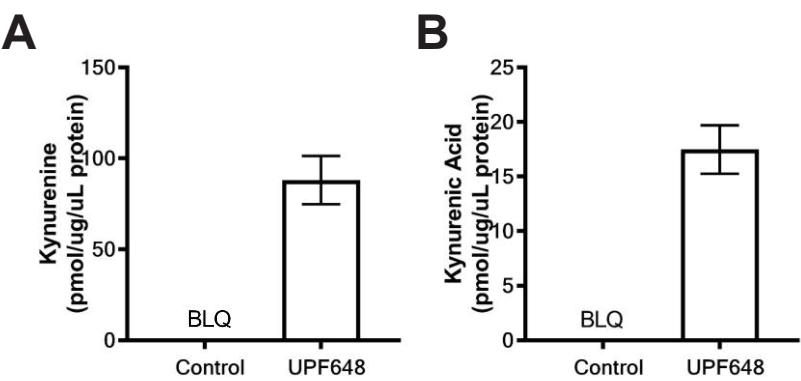

Supplement: Supplementary file 4 — Figure S4: Kmo inhibition by UPF648 leads to an accumulation of the upstream metabolites. Zebrafish larvae were treated with UPF648 or EtOH control during the hatching period (48 hpf) and for the following 48 h, the compounds were administered via the ERM and the larval development was monitored up until 96 hpf. Quantification of the metabolites upstream of Kmo: (A) kynurenine and (B) kynurenic acid after Kmo inhibition. BLQ = Below level of quantification. [file FSB2-39-e71228-s002.pdf]

Figure S5

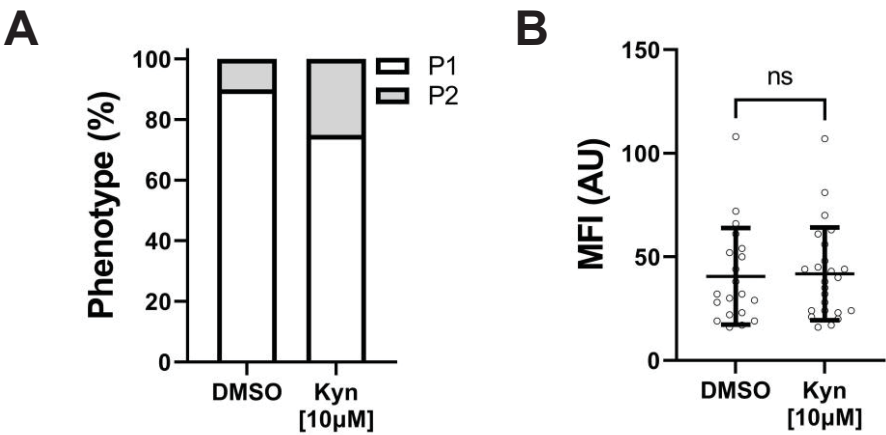

Supplement: Supplementary file 5 — Figure S5: An additional low dose of kynurenine is not sufficient to induce a renal phenotype in zebrafish larvae. Hatched zebrafish larvae were treated either with kynurenine at a concentration of 10 μM or with DMSO starting at 48 hpf, and for the following 48 h. The treatment was delivered via the embryo rearing media (ERM). Larval development was monitored until 96 hpf, at this time, readouts of edema formation and proteinuria were collected. (A) Kynurenine treatment at this concentration does not increase the proportion of larvae that develop mild to severe edema (P2‐P4). (B) Additionally, quantification of the MFI shows that kynurenine treatment at 10 μM does not lead to proteinuria (n > 10 fish for all groups; bars represent the mean intensity for each group, each circle shows the maximum fluorescence for one individual fish; comparison between two groups was done by unpaired Student t‐test with Welch's correction, n.s., nonsignificant). [file FSB2-39-e71228-s008.pdf]

Figure S6

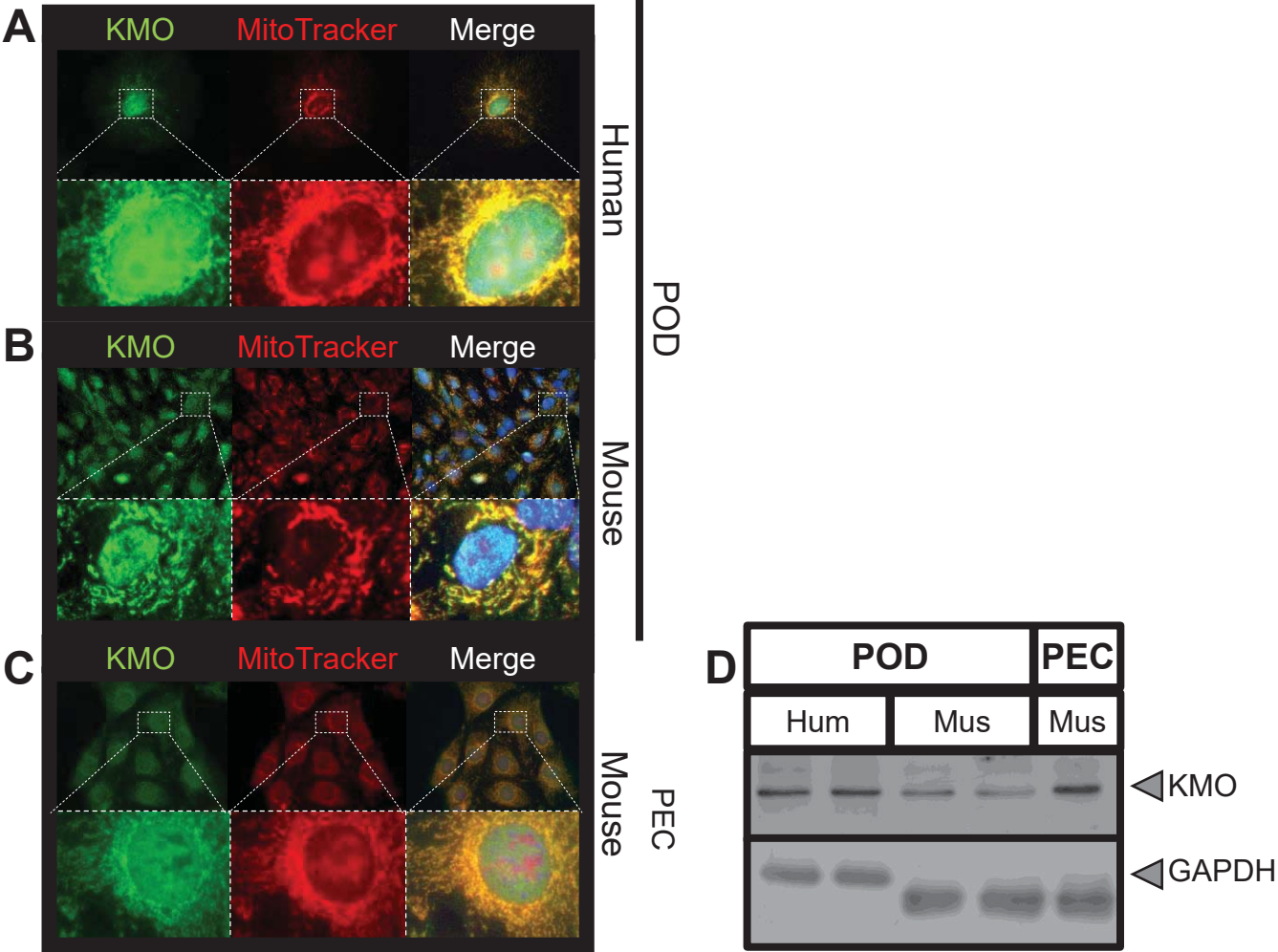

Supplement: Supplementary file 6 — Figure S6: KMO is expressed in cultured glomerular cells and it colocalizes with mitochondria. Human (A) and mouse (B) Immortalized podocytes (POD), as well as (C) murine parietal epithelial cells (PEC) express the enzyme KMO under cell culture conditions. Cells were seeded on glass cover slides and stained with an anti‐KMO antibody (green). Mitochondria were labeled using a MitoTracker fluorescent probe (red). The nuclei are visualized using DAPI (blue). The merged image shows a perinuclear expression pattern and colocalization of KMO with mitochondria, as expected. (D) Western blot of podocyte and parietal epithelial cell lysates shows KMO as a band at the expected size of 55 kDa. [file FSB2-39-e71228-s004.pdf]

**Figure S7**

**530nm**

**590nm**

**merge**

**FCCP**

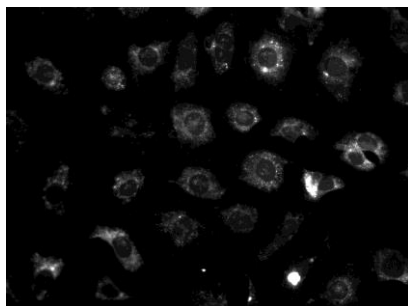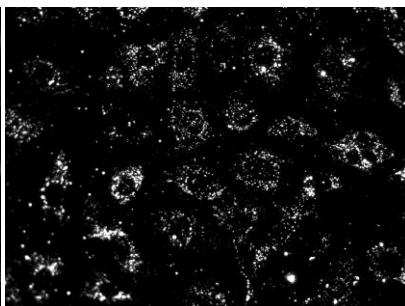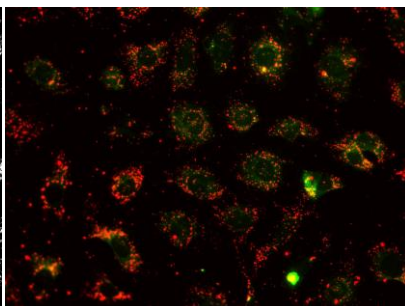

**Control**

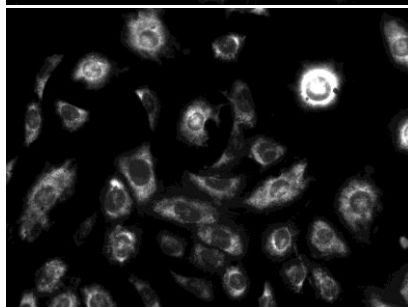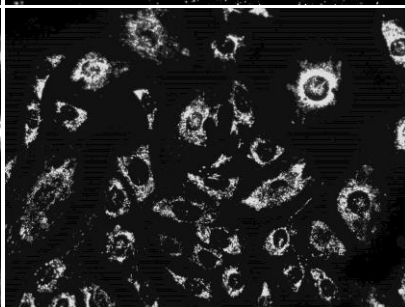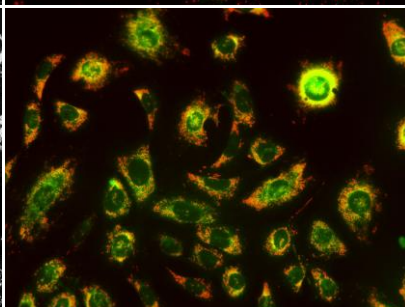

**UPF648**

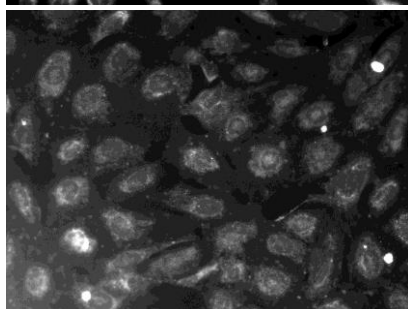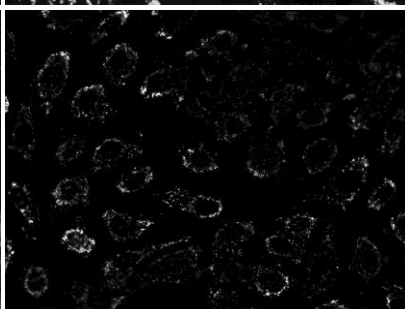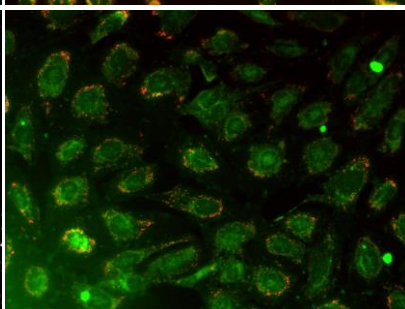

Supplement: Supplementary file 7 — Figure S7: KMO inhibition leads to mitochondrial depolarization. Mouse podocytes were treated with the KMO inhibitor UPF648. JC‐1 dye was used to visualize changes in mitochondrial membrane polarization status, showing an increase in mitochondrial depolarization. Representative images taken at 20X show single channels at 530 nm (green) and 590 nm (red), as well as merged (last column). FCCP was used as a positive control to show loss of mitochondrial membrane potential. [file FSB2-39-e71228-s001.pdf]

Figure S8

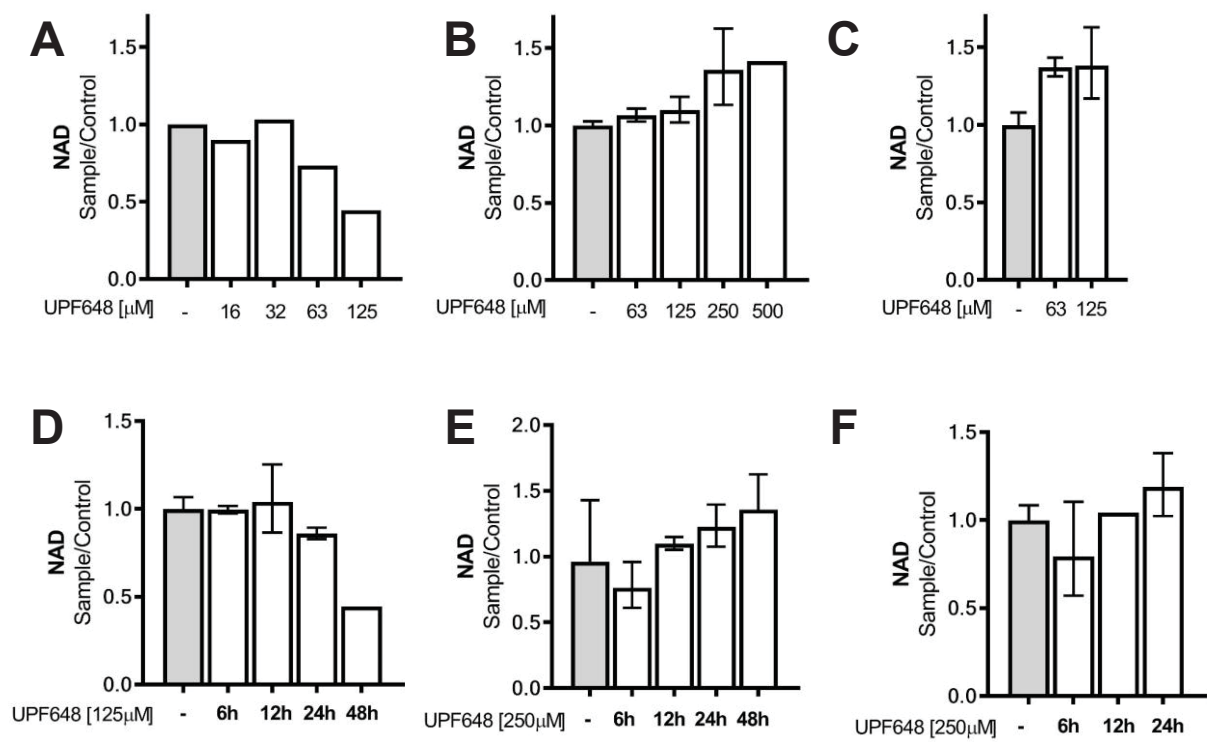

Supplement: Supplementary file 8 — Figure S8: KMO inhibition shows a difference in total NAD content in response to UPF648 treatment. Glomerular cells were treated with UPF648 at the indicated concentrations and for the specified time points. Cells were lysed after 6, 12, 24 and 48 h of KMO inhibition and were prepared for total NAD quantification by mass spectrometry. NAD quantification after treatment with UPF648 for 48 h (A) human podocytes, (B) mouse podocytes, (C) mouse parietal epithelial cells. NAD quantification after a time course of treatment with UPF648 (D) human podocytes, (E) mouse podocytes, (F) mouse parietal epithelial cells. Values were normalized by total protein content in each sample. Bars indicate the geometric mean of this ratio ± geometric SD, values represent data from 2 independent samples per time point, presented as relative to control. [file FSB2-39-e71228-s006.pdf]
